# Probing transcription factor combinatorics in different promoter classes and in enhancers

**DOI:** 10.1186/s12864-018-5408-0

**Published:** 2019-02-01

**Authors:** Jimmy Vandel, Océane Cassan, Sophie Lèbre, Charles-Henri Lecellier, Laurent Bréhélin

**Affiliations:** 1 LIRMM, Univ. Montpellier, CNRS, Montpellier, France; 2 IBC, CNRS, Univ. Montpellier, Montpellier, France; 3 IMAG, Univ. Montpellier, CNRS, Montpellier, France; 4 Institut de Génétique Moléculaire de Montpellier, University of Montpellier, CNRS, Montpellier, France; 5 Univ. Paul Valery Montpellier, Montpellier, France

**Keywords:** Regulatory genomics, Computational biology, Transcription factors, Promoters, Enhancers, mRNA, lncRNA, miRNA

## Abstract

**Background:**

In eukaryotic cells, transcription factors (TFs) are thought to act in a combinatorial way, by competing and collaborating to regulate common target genes. However, several questions remain regarding the conservation of these combinations among different gene classes, regulatory regions and cell types.

**Results:**

We propose a new approach named TFcoop to infer the TF combinations involved in the binding of a target TF in a particular cell type. TFcoop aims to predict the binding sites of the target TF upon the nucleotide content of the sequences and of the binding affinity of all identified cooperating TFs. The set of cooperating TFs and model parameters are learned from ChIP-seq data of the target TF. We used TFcoop to investigate the TF combinations involved in the binding of 106 TFs on 41 cell types and in four regulatory regions: promoters of mRNAs, lncRNAs and pri-miRNAs, and enhancers. We first assess that TFcoop is accurate and outperforms simple PWM methods for predicting TF binding sites. Next, analysis of the learned models sheds light on important properties of TF combinations in different promoter classes and in enhancers. First, we show that combinations governing TF binding on enhancers are more cell-type specific than that governing binding in promoters. Second, for a given TF and cell type, we observe that TF combinations are different between promoters and enhancers, but similar for promoters of mRNAs, lncRNAs and pri-miRNAs. Analysis of the TFs cooperating with the different targets show over-representation of pioneer TFs and a clear preference for TFs with binding motif composition similar to that of the target. Lastly, our models accurately distinguish promoters associated with specific biological processes.

**Conclusions:**

TFcoop appears as an accurate approach for studying TF combinations. Its use on ENCODE and FANTOM data allowed us to discover important properties of human TF combinations in different promoter classes and in enhancers. The R code for learning a TFcoop model and for reproducing the main experiments described in the paper is available in an R Markdown file at address https://gite.lirmm.fr/brehelin/TFcoop.

**Electronic supplementary material:**

The online version of this article (10.1186/s12864-018-5408-0) contains supplementary material, which is available to authorized users.

## Background

Transcription factors (TFs) are regulatory proteins that bind DNA to activate or repress target gene transcription. TFs play a central role in controlling biological processes, and are often mis-regulated in diseases [1]. Technological developments over the last decade have allowed the characterization of binding preferences for many transcription factors both in vitro [2, 3] and in vivo [4]. The current view is that TF combinations underlie the specificity of eukaryotic gene expression regulation [5], with several TFs competing and collaborating to regulate common target genes. As reviewed in Morgunova et al. [6] and Reiter et al. [7], multiple mechanisms can lead to TF cooperation. In its simplest form, cooperation involves direct TF-TF interactions before any DNA binding. But cooperation can also be mediated through DNA, either with DNA providing additional stability to a TF-TF interaction [8], or even without any direct protein-protein interaction. Different mechanisms are possible for the later. For example, the binding of one TF may alter the DNA shape in a way that increases the binding affinity of another TF [6]. Another system is the pioneer/settler hierarchy described in Sherwood et al. [9], with settler TFs binding DNA only if adequate pioneer TFs have already bound to open the chromatin. Lastly, other authors have hypothesized a non-hierarchical cooperative system, with multiple concomitant TF bindings mediated by nucleosomes [10]. This is related to the “billboard” system proposed for enhancers [11]. On the other hand, TFs that belong to the same protein family usually share identical or similar motifs and may compete for sites that match both motifs [12].

Several papers have studied the combinatorics of TFs from a statistical point of view. Most works aim to identify co-occurring TF pairs, i.e. pair of TFs showing binding sites that are in closest proximity than one would expect by chance. These analyses have been done either on the basis of TF binding sites (TFBSs) predicted in silico [13, 14] or with TFBSs obtained from ChIP-seq experiments [15, 16]. Depending on the approach, different difficulties may arise. In silico predicted TFBSs are known to include large amount of false positives (see below), which may bias the analyses and impede the discovery of co-occurring TFBSs. On the other hand, studies based on ChIP-seq data require as many ChIP-seq data as the number of studied TFs, and hence are intrinsically limited by the availability of these data. Moreover, with hundreds (or even thousands) of sequences, a small co-occurrence tendency may be statistically significant, even if the effect is actually very weak and would not be biologically relevant. A few works have studied TF combinations in a more global way, above the TF pair level. For example, Teng et al. [17] have applied the “frequent itemset” methodology to identify sets of co-occurring TFBSs on the basis of ChIP-seq data. However, many questions remain on the molecular determinants orchestrating TF binding and combinations [18]. Notably, with the expanding coding capacity of the human genome [19, 20], it remains to determine whether the expression of all gene classes, in particular coding mRNAs, long non-coding(lnc)RNAs and micro(mi)RNAs, is controlled by similar TF combinations in a given cell type. Likewise, TFs control gene expression through the binding of promoters and enhancers, which harbor similar but also specific genomic features [21]. It is then not clear whether the binding preferences of a given TF are similar in enhancers and promoters.

Here, we analyze global TF combinations from a different perspective. Rather than identifying TF pairs/sets that co-occur more frequently than expected by chance, we aim to identify TF combinations that can be predictive of the binding of a target TF. More formally, given a class of regulatory sequences (for example 500 bp around the TSSs of the coding genes) and a ChIP-seq experiment targeting a specific TF in a specific cell type, we aim to identify the combinations of TFs whose predicted TFBSs can be used for predicting which sequences are effectively bound by the target TF in this cell type. Hence, rather than using purely statistical co-occurrence analysis, we study TF combinations in the framework of a TFBS prediction problem. The approach has several advantages. First, a single ChIP-seq experiment is theoretically sufficient to identify all TFs cooperating/competing with the target TF in the target cell type. Next, if a TF is selected in the combination, this means that its predicted binding sites are indicative of the presence of the target TF, which limit the number of false positives and the problems of spurious statistical significances. Finally, the approach takes into account all TFs and can therefore identify all possible TF combinations not just TF pairs.

TFBSs are traditionally modeled with position weight matrices (PWMs) [22]. Several databases such as JASPAR [23], HOCOMOCO [24], CisBP [25] and Transfac [26], propose position frequency matrices (PFM, which can be transformed in PWMs) for hundred of TFs. These PWMs can be used to scan sequences and identify TFBSs using tools such as FIMO [27] or MOODS [28]. However, while a PWM usually identifies thousands of potential binding sites for a given TF in the genome [29], ChIP-seq analyses have revealed that only a fraction of those sites are effectively bound [30]. There may be different reasons for this discrepancy between predictions and experiments. First, PWMs implicitly assume that the positions within a TFBS independently contribute to binding affinity. Several approaches have thus been proposed to account for positional dependencies within the TFBS (see for example [31, 32]). Other studies have focused on the TFBS genomic environment, revealing that TFs positions of their core binding sites [33, 34]. Beyond the primary nucleotide sequence, structural constraints may also affect TF binding. For example, it is thought that TFs use DNA shape features to distinguish binding sites with similar DNA sequences [35, 36]. Some attempts have thus been made to integrate DNA shapes information with PWMs [37, 38]. Other studies have investigated the link between TF binding and epigenetic marks, showing that many TFs bind regions associated with specific histone marks [39]. Similarly, ChIP-seq experiments also revealed that most TFBSs fall within highly accessible (i.e., nucleosome-depleted) DNA regions [40]. Consequently, several studies have proposed to supplement PWM information with DNA accessibility data to identify the active TFBSs in a given cell type [41–43]. However, it remains unclear whether these chromatin states are a cause or a consequence of TF binding [44]. Hence, while these approaches may be very informative for predicting TF binding, they should be used with caution if the goal is also to identify the DNA determinants of the binding. Besides, these approaches do not take into account TF combinations, which, as already discussed, may be important determinants of TF binding. For this reason, studying TF combinations through a TFBS prediction problem appears as an appealing approach.

It is important to note that beyond approaches based on known PWMs, several ab initio methods have also been proposed recently for predicting TFBSs from raw data sequences. Notably, deep learning approaches based on neural networks have proved to give higher prediction accuracy than simple PWM-based methods [45, 46]. However, ab initio methods, and particularly neural network approaches, are difficult to interpret (the inherent trade-off between accuracy and interpretability). Although some attempts have been made to post-analyze learned neural networks (see for example [47]), studying TF combinations and DNA determinants of TF binding from these models is not straightforward.

Hence, we devised a simple non ab initio strategy names TFcoop that predicts if a target TF binds a sequence of interest using two kinds of variables: i) the binding affinity (i.e. PWM affinity score) of the target TF as well as any other TF identified as cooperating with the target TF; and ii) the nucleotide composition of the sequence. TFcoop is based on a logistic model. The set of cooperating TFs and the model parameters are learned from ChIP-seq data of the target TF via LASSO penalization [48]. Learning can be done using a moderate amount of data, which allows us to learn specific models for different types of regulatory sequences. Using ChIP-seq data from the ENCODE project, we applied TFcoop to investigate the TF combinations involved in the binding of 106 different TFs on 41 different cell types and in four different regulatory regions: promoters of mRNAs, lncRNAs and miRNAs, and enhancers [19, 20, 49, 50]. We first showed that the approach outperforms simple PWM methods and has surprisingly good accuracy, close to that of ab initio methods like DeepSea [45]. We next assessed with independent experimental data that the cooperative TFs predicted by TFcoop actually bind the same regulatory sequences as the target TF. Then, we used TFcoop to analyze TF combinations in different cell types and regulatory regions. First, we show that TF combinations governing the binding of the target TF on promoters are similar for different cell-types but distinct in the case of enhancer binding. Second, for a given TF, we observe that TF combinations are different between promoters and enhancers, but similar for promoters of all gene classes (mRNAs, lncRNAs, and miRNAs). Analysis of the composition of TFs cooperating with the different targets show over-representation of pioneer TFs [9], especially in promoters, as well as binding sites with nucleotide composition similar to that of the target TF. We also observed that cooperating TFs are enriched for TFs whose binding is weakened by methylation [51]. Lastly, our models can accurately distinguish promoters into classes associated with specific biological processes.

## Results

### Computational approach

Given a target TF, the TFcoop method identifies the TFBS combination that is indicative of the TF presence in a regulatory region. We first considered the promoter region of all mRNAs (defined as the 1000bp centered around gene start). TFcoop is based on a logistic model that predicts the presence of the target TF in a particular promoter using two kinds of variables: PWM affinity scores and (di)nucleotide frequencies (see “Methods” section). For each promoter sequence, we computed the affinity score of the 638 JASPAR PWMs (redundant vertebrate collection for 519 different TFs), and the frequency of every mono- and dinucleotide in the promoter. These variables were then used to train a logistic model that aims to predict the outcome of a particular ChIP-seq experiment in mRNA promoters. Namely, every promoter sequence with a ChIP-seq peak is considered as a positive example, while the other sequences are considered as negative examples (see below). In the experiments below, we used 409 ChIP-seq datasets from ENCODE and different models. Each model targets one TF and one cell type. Given a ChIP-seq experiment, the learning process involves selecting the PWMs and (di)nucleotides that can help discriminate between positive and negative sequences (this is done by way of the LASSO penalization [48]), and estimate the model parameters that minimize prediction error. Note that the learning algorithm can select any predictive variable including the PWM of the target TF. See “Methods” section for more details on the data and logistic model. Note also that, while several classification approaches are available in the literature, all methods are not suitable for our problem. Because our aim is to identify TF combinations, only methods implementing a feature selection procedure are eligible. To this aim, LASSO penalization is often considered as a method of choice [52]. An alternative would be to use classification trees, but this method is known to suffer from stability issues [53].

We used two different procedures for selecting the positive and negative sequences. Each procedure actually defines a different classification problem. In the first case, we kept all positive sequences (i.e. promoters overlapping a ChIP-seq peak in the considered ChIP-seq experiment), and randomly selected the same number of negative sequences among all sequences that do not overlap a ChIP-seq peak. In the second case, we used an additional dataset that measures gene expression in the same cell type as the ChIP-seq data. We then selected all positive sequences with non zero expression level and randomly selected the same number of negative sequences among all sequences that do not overlap a ChIP-seq peak but that have a similar expression level as the selected positive sequences. Hence, in this case (hereafter called the expression-controlled case), we learn a model that predicts the binding of a target TF in a promoter knowing that the corresponding gene is expressed. On the contrary, in the first case we learn a model that predicts the binding without knowledge about gene expression. The purpose of the expression-controlled case is to decipher TF combinations independently of the effect of epigenetic modifications that are linked to expression (e.g. DNA methylation and various histone marks). As all selected sequences are associated with expressed genes, the positives and negatives sequences are likely to be associated with the same epigenetic marks.

### TFcoop assessment

**Fig. 1:**
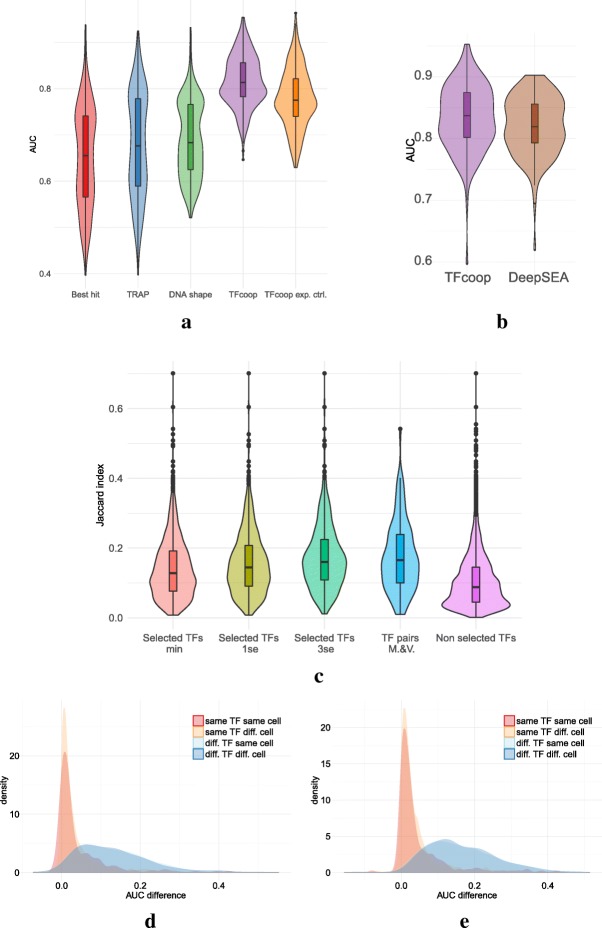
Accuracy and specificity on mRNA promoters. **a** Violin plots of the area under the ROC curves obtained in the 409 ChIP-seq. Best hit (red), TRAP (blue), DNAshape (green), TFcoop with no expression control (purple), and TFcoop with expression control (orange). ROC curves for Best hit, TRAP and DNAshape were computed in the non expression-controlled case. **b** Comparison of AUC achieved by TFcoop and DeepSea approach [45]. Comparison was done on 214 ChIP-seq experiments for which the DeepSea server provides predictions. **c** Intersection between pairs of ChIP-seq experiments associated with TFs identified as cooperating in promoters. These violin plots report the distribution of Jaccard indexes computed between different pairs of Chip-seq experiments. Red, olive and green: for each TF A, we measured the Jaccard index between promoters bound by A and promoters bound by a TF B whose PWM has been selected in the TFcoop model learned for A (cases B = A were not considered). *λ*_min_, *λ*_1se_ and *λ*_3se_ denote three inclusive sets of parameters of increasing importance (see Methods for details). Purple: for each TF A, we measured the Jaccard index between promoters bound by A and promoters bound by TFs whose PWMs have not been selected in the A model. Blue: for each TF pair A-B identified in [13] (Additional file 1: Figure S1), we measured the Jaccard index between promoters bound by A and promoters bound by B. Samples red, olive, green, blue and purple have been computed from 2796, 1723, 1037, 282 and 14,529 pairs, respectively. **d**–**e** Distribution of AUC differences obtained when using a model learned on a first ChIP-seq experiment to predict the outcome of a second ChIP-seq experiment. Different pairs of ChIP-seq experiments were used: experiments on the same TF and same cell type (red), experiments on the same TF but different cell types (yellow), experiments on different TFs but same cell type (light blue), and experiments on different TFs and different cell types (blue). For each pair of ChIP-seq experiment A-B, we measured the difference between the AUC achieved on A using the model learned on A, and the AUC achieved on A using the model learned on B. AUC differences were measured on the non expression-controlled case (**d**) and on the expression-controlled case (**e**)

We ran TFcoop on the 409 ChIP-seq datasets and for the two prediction problems. The accuracy of each model was assessed by cross-validation by plotting the Receiver Operating Curve (ROC) and measuring the Area Under the Curve (AUC). For comparison, we also measured the accuracy of the classical approach that discriminates between positive and negative sequences using only the affinity score of the PWM associated with the target TF. In addition, we estimated the accuracy of the TRAP method, which uses a biophysically inspired model to compute PWM affinity [54] and that of the approach proposed in [37], which integrates DNA shape information with PWMs. As shown in Fig. 1a and Additional file 1: Figure S1 and Figure S2, TFcoop outperforms these PWM-based approaches on many TFs (t-test *p*-values 1.4*e*^−106^, 2.2*e*^−104^ and 7.1*e*^−80^). Note that these comparisons are rather unfavourable for our method because they integrate all 69 CTCF experiments, while TFcoop has similar accuracy than classical PWM methods on this TF (see Additional file 1: Figure S2). Concerning TFcoop accuracy, we can observe a strong link between the number of training sequences and the AUC (see Additional file 1: Figure S3). Next, we ran TFcoop with tri- and quadri-nucleotide frequencies in addition to di-nucleotide frequencies. Although a consistent AUC improvement was observed, the increase was very slight most of the time (Additional file 1: Figure S4). Similarly, we also ran TFcoop on two alternative PWM libraries that both involve slightly more TFs than the JASPAR library (CisBP [25] and HOCOMOCO [24], see “Methods” section) but we observed similar results as that obtained with JASPAR (Additional file 1: Figure S5). Lastly, we compared TFcoop accuracy to that of the deep learning approach DeepSea [45] and observed very close results (see Fig. 1b; t-test *p*-value 0.048). Hence, TFcoop performances appear to be in the range of that of classical ab initio methods.

Next, we sought to assess the TF cooperations inferred by the models. If true, they should be apparent in the ChIP-seq experiments. Namely, if the PWM of TF B is among the selected variables for predicting the presence of TF A, then we should observe many common targets among the ChIP-seq experiments of TFs A and B. To test this, we first randomly selected one model for each different TF, and restricted our analyses to the PWMs associated with TFs with ENCODE ChIP-seq experiments. Then, for each model A, we measured the Jaccard index between promoters bound by TF A and promoters bound by a TF B whose PWM has been selected in model A (cases B = A were not considered), and we compared these scores to the same scores computed on TFs whose PWMs have not been selected in model A (see “Methods” section). The LASSO procedure allows us to rank the selected variables from the most predictive to the less predictive ones. We measured the Jaccard index for different cutoffs in this ranked list and observed that Jaccard indexes i) vary accordingly with the cutoff and ii) are always larger than Jaccard indexes computed for non-selected TFs (t-test *p*-values <1.*e*^−16^; see Fig. 1c). Hence, the inferred TF cooperations are supported by experimental data. For comparison purpose, we repeated the same analysis with TF pairs identified by Myšičková and Vingron (2012) and found very similar performance measures (see Fig. 1c).

Finally, we sought to take advantage of the relative redundancy of target TFs in the set of 409 ChIP-seq experiments to investigate the specificity of the learned models. Namely, we compared pairs of models learned from ChIP-seq experiments targeting (i) the same TF in the same cell-type, (ii) the same TF in different cell-types, (iii) different TFs in the same cell-type, and (iv) different TFs in different cell-types. In these analyses, we used the model learned on one ChIP-seq experiment A to predict the outcome of another ChIP-seq experiment B, and we compared the results to those obtained with the model directly learned on B. More precisely, we measured the difference of AUC between the model learned on A and applied on B and the model learned and applied on B. To avoid any effect driven by the over-representation of CTCF in ChIP-seq data, we randomly selected only 10 ChIP-seq experiments targeting this TF in these analyses. As shown in Fig. 1d and e, models learned on the same TF (whether or not on the same cell-type) have overall smaller AUC differences than models learned on different TFs. For sake of comparison, we also ran the same analysis on non-ENCODE ChIP-seq data targeting 10 different TFs (see “Methods” section). Namely, we used the models learned on the corresponding ENCODE data to predict the outcome of these 10 non-ENCODE data. Results are overall very similar to those obtained on ENCODE data (median AUC 0.83 on ENCODE data vs. 0.82 on non-ENCODE data; see Additional file 1: Figure S6).

We then analyzed cell and TF specificity more precisely. Cell specificity refers to the ability of a model learned on one TF and in one cell type to predict the outcome of the same TF in another cell type. Oppositely, TF specificity refers to the ability of a model learned on one TF in one cell type to predict the outcome of another TF in the same cell type. Cell and TF specificities were evaluated by the shift between the associated distributions of AUC differences in Fig. 1d: cell specificity was assessed by the shift between red and yellow distributions, while TF specificity was assessed by the shift between red and light blue distributions. We used a standard t-test to measure that shift. Low *p*-values indicate high distribution shifts (hence high cell/TF specificity), while high *p*-values indicate low shifts (hence low specificity). Our results indicate very low cell specificity (*p*-values 0.91 and 0.95 in the non-controlled and expression-controlled cases, respectively) and high TF specificity (1·10^−61^ and 3·10^−83^). The fact that the TF specificity is slightly higher in the expression-controlled case suggests that part of the TF combinations that help discriminate between bound and unbound sequences is common to several TFs in the non-controlled case. It is indeed known that the majority of ChIP-seq peaks are found in open and active promoters [40]. Thus, most positive examples are associated with open chromatin marks. However, in the non-expression-controlled case, a large part of the negative examples are in closed chromatin and are therefore likely associated with other chromatin marks. As a result, in this case, TFcoop presumably also learns the TFBS signature that helps differentiate between these chromatin marks. Oppositely, in the expression-controlled case, the positive and negative examples have similar chromatin states, and TFcoop unveils the TFBS signature specific to the target TF. We can also observe that this renders the former problem slightly easier than the second one, as illustrated by the difference of TFcoop performances in Fig. 1a (t-test *p*-value 2.6*e*^−18^). Finally the low cell specificity means that the general rules governing TFBS combination in promoters do not dramatically change from one tissue to another. This is important in practice because it enables us to use a model learned on a specific ChIP-seq experiment to predict TBFSs of the same TF in another cell type.

### Analysis of TFBS combinations in promoters

We next analyzed the different variables (PWM scores and (di)nucleotide frequencies) that were selected in the 409 learned models. Overall, 95% of the variables correspond to PWM scores. Although only 5% of the selected variables are (di)nucleotide frequencies, almost all models include at least one of these features. As mentioned earlier, the learning algorithm does not use any prior knowledge and can select the variables that best help predict the ChIP-seq experiment without necessarily selecting the PWM of the target TF. Our analysis shows that, for 75% of the models, at least one version of the target PWM was selected. Moreover, it is important to note that similar PWMs tend to have correlated scores. Hence, another PWM may be selected instead of the target. To overcome this bias, we also considered all PWMs similar to the target PWM. We used Pearson correlation between PWM scores in all promoters to measure similarity and set a threshold value of 0.75 to define the list of similar PWMs. With this threshold, 90% models include the target or a similar PWM. Analysis of the remaining 10% models shows that they often correspond to ChIP-seq experiments with low number of positive sequences (median number 955 vs. 2477 for all ChIP-seq experiments). This may be due either to technical problems, to lowly expressed TFs, or to TFs that rarely bind promoters.

Next we thought to investigate the contribution alternative PWMs may have on model performance. For this, we ran a whole new analysis using the non-redundant JASPAR PWM library (one PWM per TF, i.e. 519 PWMs). As shown in Additional file 1: Figure S5, results are slightly less accurate than with the complete (redundant) database, illustrating the fact that alternative motifs provide important information unveiled by TFcoop.

We further analyzed the most selected PWMs. To avoid any bias linked to the number of CTCF ChIP-seq experiments, we only considered 10 CTCF models that were randomly selected for the analyses. We ranked the PWMs by the number of models in which they appear, and look for enrichment of certain JASPAR structural families (bHLH, Zinc finger, …). A gene set enrichment analysis (GSEA, see “Methods” section) [55] shows that “tryptophan cluster factors” (FDR q-val <10^−4^), “C2H2 zinc finger factors” (FDR q-val <10^−4^) and “basic leucine zipper factors” (FDR q-val =2·10^−3^) are the most represented classes of PWMs selected in the models (Additional file 1: Figure S7). We then looked at the differences between models learned in the expression-controlled experiments and models learned in the non-controlled experiments. For each non-controlled model, we enumerated the variables that are selected in this model and not selected in the corresponding expression-controlled model. Several PWMs are over-represented in this list (see Additional file 1: Table S1).

**Fig. 2:**
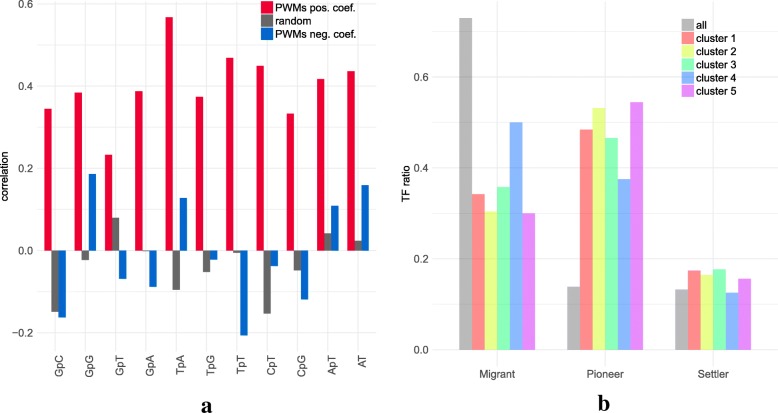
Selected PWMs in mRNA promoters. **a** Pearson correlation between nucleotide composition of the target PWM and the mean composition of selected PWMs (with positive and negative coefficients in red and blue, respectively) in 409 models. Grey: correlation achieved by randomly selecting the same number of PWMs for each model. **b** Pioneer TF distribution of selected PWMs in the different models. We kept one model for each target PWM to avoid bias due to over-representation of the same PWM in certain classes. Grey represents the distribution of all PWMs associated with a family in Sherwood et al. [9] (159 over 519 non-redundant PWMs)

Next, following the analyses of Levo et al. [33] and Dror et al. [34] we used our models to investigate the link between the nucleotide composition of the target PWM and that of the TFBS flanking region. First, we did not observe a significant link between target PWM composition and the (di)nucleotide variables that were selected in the models (Kolmogorov-Smirnov test *p*-val =0.448; see Additional file 1: Figure S8). However, the (di)nucleotide composition of target PWM exhibited strong resemblance to that of the other selected PWMs (see Fig. 2a). Specifically, the nucleotide and dinucleotide frequencies of the target PWM were strongly positively correlated with that of the PWMs selected with a positive coefficient. For PWMs selected with a negative coeficient the correlations are moderate or negative. This is in accordance with the findings of Dror et al. [34], who show that TFBS flanking regions often have similar nucleotide composition as the the TFBS.

We next evaluated the possibility of clustering the 409 learned models using the selected variables. As shown in Additional file 1: Figure S10, the models can be partitioned in a few different classes with a k-means algorithm (5 classes were used in this figure). Additional file 1: Figure S11 reports the most used variables in these different classes. We can first observe that, in agreement with our analysis of model specificity, the models associated with the same TF tend to cluster together. For example, the 4^*th*^ class of our clustering (the blue one in Additional file 1: Figure S10) is exclusively composed of CTCF models. Note that we did not observe any enrichment for the classical TF structural families (bHLH, Zinc finger, …) in the different classes (data not shown). Actually, the clustering seems to be essentially driven by the nucleotide composition of the PWMs belonging to the models (see Additional file 1: Figure S12).

Pioneer TFs are thought to play an important role in transcription by binding to condensed chromatin and enhancing the recruitment of other TFs [9]. As shown in Fig. 2b and by a GSEA analysis (Additional file 1: Figure S9), pioneer factors clearly are over-represented in the selected variables of the models, whereas they represent less than 14% of all TFs. These findings are in agreement with their activity: pioneer TFs occupy previously closed chromatin and, once bound, allow other TFs to bind nearby [9]. Hence the binding of a given TF requires the prior binding of at least one pioneer TF. We also observed that TFs whose binding is weakened by methylation [51] are enriched in all models (Additional file 1: Figure S13). This result may explain how CpG methylation can negatively regulate the binding of a given TF in vivo while methylation of its specific binding site has a neutral or positive effect in vitro [51]: regardless of the methylation status on its binding site, the binding of a TF can also be influenced in vivo by the sensitivity of its partners to CpG methylation.

### TFBS combinations in lncRNA and pri-miRNA promoters

**Fig. 3:**
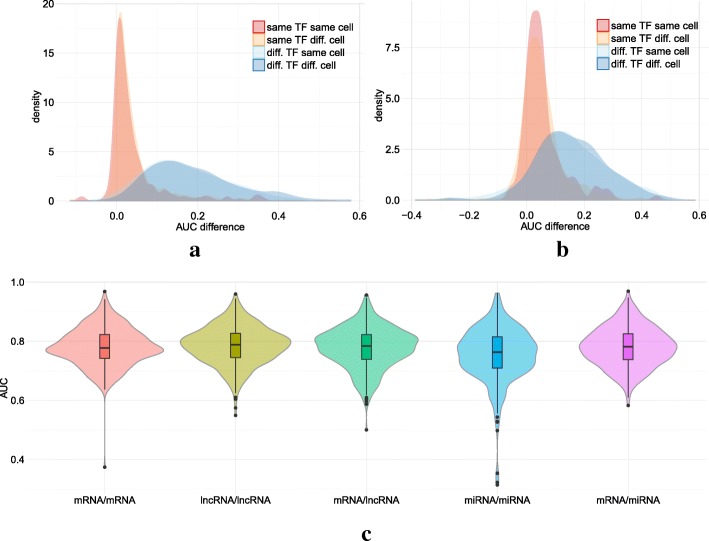
Accuracy and specificity on lncRNA and pri-miRNA promoters. Top: Model specificity on promoters of lncRNA (**a**) and pri-miRNAs (**b**). These figures represent the distribution of AUC differences obtained when using a model learned on a first ChIP-seq experiment to predict the outcome of a second ChIP-seq experiment. Different pairs of ChIP-seq experiments were used: experiments on the same TF and same cell type (red), experiments on the same TF but different cell types (yellow), experiments on different TFs but same cell type (light blue), and experiments on different TFs and different cell types (blue). For each pair of ChIP-seq experiment A-B, we measured the difference between the AUC achieved on A using the model learned on A, and the AUC achieved on A using the model learned on B. AUC differences were measured on the expression-controlled case. Bottom: Promoter models are interchangeable. For each ChIP-seq experiment, we computed the AUC of the model learned and applied on mRNAs (pink), learned and applied on lncRNAs (yellow-green), learned and applied on pri-miRNAs (blue), learned on mRNAs and applied to lncRNAs (green), learned on mRNAs and applied to pri-miRNAs (purple)

We then ran the same analyses on the promoters of lncRNAs and pri-miRNAs using the same set of ChIP-seq experiments. Results are globally consistent with what we observed on mRNA promoters (see Fig. 3 for the expression-controlled case). Overall, models show good accuracy and specificity on lncRNAs. Models are less accurate and have lower specificity for pri-miRNAs but this likely results from the very low number of positive examples available for these genes in each ChIP-seq experiment (Additional file 1: Figure S14), which impedes both the learning of the models and estimation of their accuracy.

Next we sought to compare the models learned on mRNA promoters to the models learned on lncRNA and pri-miRNA promoters. For this, we interchanged the models learned on the same ChIP-seq experiment, i.e. we used the model learned on mRNA promoters to predict the outcome on lncRNA and pri-miRNA promoters. One striking fact illustrated by Fig. 3c and Additional file 1: Figure S15 is that models learned on mRNA promoters and those learned on lncRNA promoters are almost perfectly interchangeable. This means that the TFBS rules governing the binding of a specific TF in a promoter are similar for both types of genes. We obtained consistent results when we used the models learned on mRNAs to predict the ChIP-seq outcomes on pri-miRNA promoters (Fig. 3c and Additional file 1: Figure S15). Accuracy is even better than that obtained by models directly learned on pri-miRNA promoters, illustrating the fact that the poor performance achieved on pri-miRNA promoters likely results from the small number of learning examples available for these genes.

### TFBS combinations in enhancers

We next applied the same approach on 38,554 enhancers defined by the FANTOM consortium [50]. We used the same ChIP-seq experiments as for the promoters. All enhancer sequences overlapping a ChIP-seq peak in the considered ChIP-seq experiment were considered as positive examples. As for promoters, we used two strategies to select positives and negative examples: in a first case we did not apply any control on their expression, while in a second case, we used CAGE expression data in the different tissues to only select expressed enhancers.

**Fig. 4:**
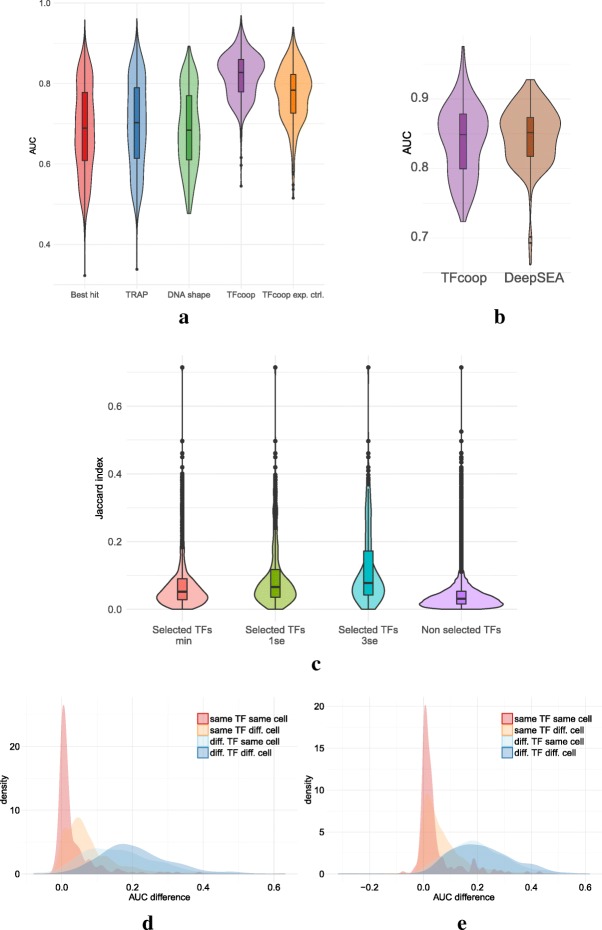
Accuracy and specificity on enhancers. **a** Violin plots of the area under the ROC curves obtained in the 409 ChIP-seq. Best hit (red), TRAP (blue), DNAshape (green), TFcoop with no expression control (purple), and TFcoop with expression control (orange). ROC curves for Best hit, TRAP and DNAshape were computed in the non expression-controlled case. **b** Comparison of AUC achieved by TFcoop and DeepSea approach [45]. Comparison was done on 214 ChIP-seq experiments for which the DeepSea server provides predictions. **c** Intersection between pairs of ChIP-seq experiments associated with TFs identified as cooperating in promoters. These violin plots report the distribution of Jaccard indexes computed between different pairs of Chip-seq experiments. Red, green and blue: for each TF A, we measured the Jaccard index between promoters bound by A and promoters bound by a TF B whose PWM has been selected in the TFcoop model learned for A (cases B = A were not considered). *λ*_min_, *λ*_1se_ and *λ*_3se_ denote three inclusive sets of parameters of increasing importance (see “Methods” section for details). Purple: for each TF A, we measured the Jaccard index between promoters bound by A and promoters bound by TFs whose PWMs have not been selected in the A model. **d**–**e** Distribution of AUC differences obtained when using a model learned on a first ChIP-seq experiment to predict the outcome of a second ChIP-seq experiment on enhancers. Different pairs of ChIP-seq experiments were used: experiments on the same TF and same cell type (red), experiments on the same TF but different cell types (yellow), experiments on different TFs but same cell type (light blue), and experiments on different TFs and different cell types (blue). For each pair of ChIP-seq experiment A-B, we measured the difference between the AUC achieved on A using the model learned on A, and the AUC achieved on A using the model learned on B. AUC differences were measured on the non expression-controlled case (**d**) and on the expression-controlled case (**e**)

As observed for promoters, TFcoop outperforms classical PWM-based approaches on many TFs (see Fig. 4a and Additional file 1: Figure S16; t-test *p* values 2.2*e*^−77^, 9.7*e*^−67^ and 1.4*e*^−88^) and achieves results close to that of DeepSea [45] (Fig. 4b, t-test *p*-value 0.37). Here again, the non expression-controlled problem seems slightly easier than the controlled one (t-test *p*-value 6.1*e*^−23^). Using the same “Jaccard index test” used for promoters, we also assessed that the TF cooperations inferred by the models can be observed in ChIP-seq data and hence are likely to be biologically valid (*p*-value <1.*e*^−16^ and Fig. 4c).

However, analysis of model specificity reveals somewhat different results from that observed for promoters. Globally, models have good TF specificity: models learned on the same TF have more similar prediction accuracy than models learned on different TFs. However, in contrast to promoters, cell specificity is high in the non-controlled case (*p*-value 2·10^−45^; see peak shift in Fig. 4d), although much lower in the expression-controlled case (*p*-value 1.6·10^−12^). Additionally, TF specificity seems slightly higher in the expression-controlled case than in the non-controlled case (*p*-values 1.7·10^−102^ vs. 1.·10^−114^). This is in accordance with our hypothesis formulated for promoters, that part of the TF combinations learned by TFcoop in the non-controlled case actually differentiates between active and inactive chromatin marks. This also seems to indicate that these TF combinations are cell-type specific, while the remaining combinations are more general (as illustrated by the 1.6·10^−12^*p*-value measured on the expression-controlled case). Moreover, analysis of selected variables reveals that models learned without expression control involve much more variables than models learned with expression control (median numbers 18 vs. 11; t-test *p*-value ∼10^−9^). As a consequence, several variables are statistically more abundant in non-controlled models than in the cognate expression-controlled models (see Additional file 1: Table S1). Interestingly, among the four variables with the most important differences, three are dinucleotides CpG, TpC and ApT. This may indicate that part of the active/inactive chromatin marks is linked to the dinucleotide composition of the underlying sequence. This proposal is in line with findings revealing the existence of sequence-level instructions for chromatin modifications [45, 46, 56]. Moreover, a GSEA analysis shows that the PWMs with the strongest differential enrichments belong to the “three-zinc finger kruppel-related factors” (FDR q-val 1·10^−2^). As some of these factors, in particular KLF1 [57], are linked to chromatin remodeling, this enrichment supports the idea that TFcoop also identifies TF combinations linked to epigenetics. The fact that cell-type specificity is more apparent for enhancers than for promoters in the non expression-controlled case (2·10^−45^ for enhancers vs. 0.91 for promoters) is in accordance with the fact that, contrary to promoters, most enhancers are expressed in a cell-specific manner (Additional file 1: Figure S17 and ref. [50]).

**Fig. 5:**
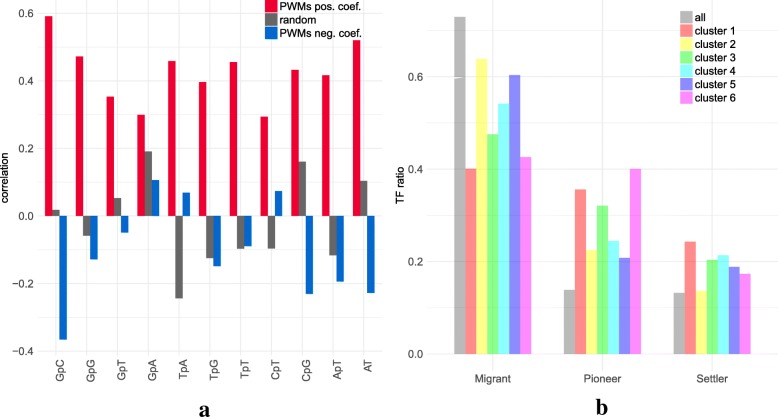
Selected PWMs in enhancers. **a** Pearson correlation between nucleotide composition of the target PWM and the mean composition of selected PWMs (see legend of Fig. 2a) **b** Pioneer TF distribution in selected PWMs (same legend as Fig. 2b)

As for promoters, we observed that the selected PWMs tends to have similar (di)nucleotide composition as the target PWM (Fig. 5a). Moreover, models can also be partitioned in a few different classes according to the selected variables (Additional file 1: Figure S18 and Additional file 1: Figure S19). These classes mostly correspond to the nucleotide composition of the target and selected PWMs (Additional file 1: Figure S20). Pioneer TFs are also over-represented in the selected PWMs (Fig. 5b and Additional file 1: Figure S9).

**Fig. 6:**
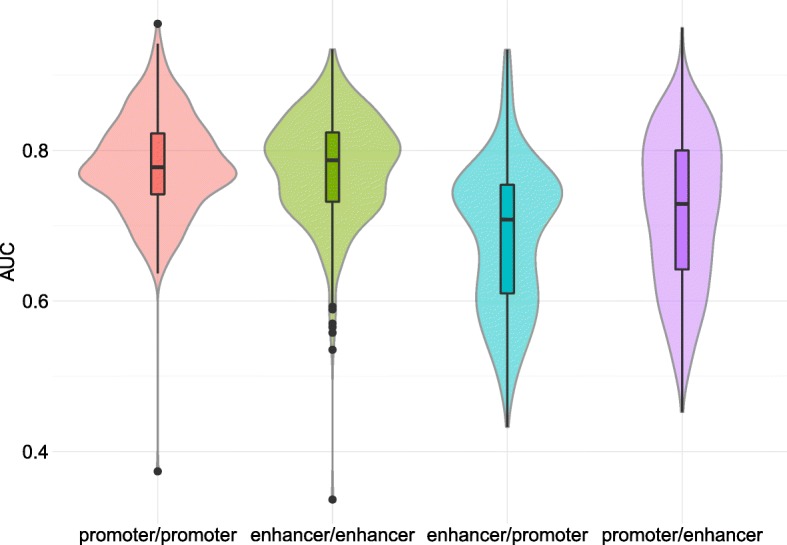
AUCs obtained in mRNA promoter and enhancer models. For each ChIP-seq experiment we computed the AUC of the model learned and applied on the promoters (red), learned and applied on the enhancers (green), learned on enhancers and applied to promoters (blue), and learned on promoters and applied to enhancers (purple)

Next we sought to compare the models learned on enhancers to the models learned on promoters. First, we observed that enhancer models involve PWMs that are different from that used in promoter models (Additional file 1: Table S2). Note for instance that several AP-1 TFs (FOS/JUN) are enriched in enhancers, in accordance with their prominent role in enhancer selection [58]. The same three structural classes are found enriched, but in different proportions, with more “C2H2 zinc finger factors” in promoters and more “basic leucine zipper factors” in enhancers (Additional file 1: Figure S7). In term of prediction, promoter and enhancer models have globally similar accuracy (see Fig. 6 on the expression-controlled cases). However, a pairwise comparison of the models learned on each ChIP-seq experiment shows that the prediction accuracy is only moderately correlated (Pearson correlation 0.33; see Additional file 1: Figure S21). Moreover, if we interchange the two models learned on the same ChIP-seq experiment, we observe that the model learned on promoters is generally not as good on enhancers as it is on promoters and *vice-versa* (Fig. 6). Hence, while the rules learned on enhancers (promoters) in a given cell type are globally valid for enhancers (promoters) of other cell types, they do not apply to promoters (enhancers) of the same cell type. Note that AUCs of models learned on promoters and applied to enhancers are greater than that of models learned on enhancers and applied to promoters (Fig. 6). This result might be explained by the existence of promoters able to exert enhancer functions [59, 60]. Conversely, the FANTOM definition of enhancers precludes potential promoter functions [50].

### Using TFcoop scores to describe regulatory sequences

**Fig. 7:**
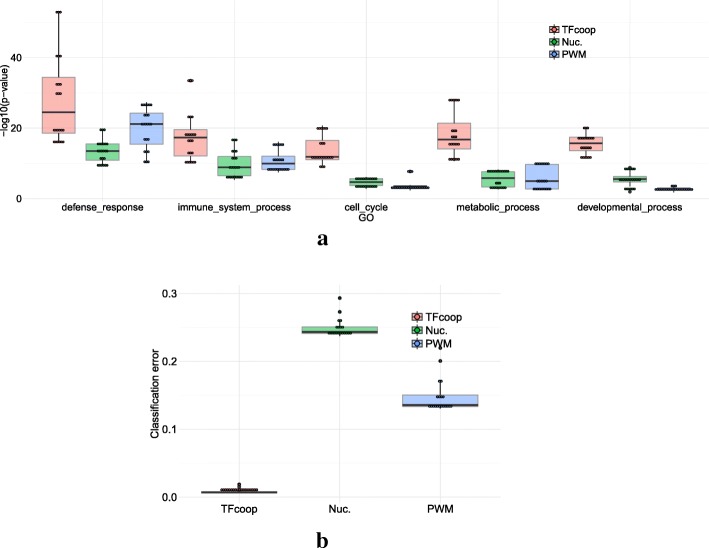
Using TFcoop scores for describing regulatory sequences. **a** GO term enrichment obtained with different promoter descriptions. Promoters were described using three different representations—TFcoop scores (red), (di)nucleotide frequencies (green), classical PWM scores (blue)— and then partitioned several times with different k-means and different class numbers (see main text). For each clustering we identified the best *p*-value (Fisher exact test) associated with 5 GO terms (“defense response”, “immune system process”, “cell cycle”, “metabolic process”, “developmental process”) in any cluster. **b** Classification errors achieved with KNN classifiers discriminating between promoter and enhancer sequences. Boxplots describe the errors obtained using TFcoop scores (red), (di)nucleotide frequencies (green), and the classical PWM scores (blue), using different number of neighbors (K)

We next explored whether TFcoop scores could be used to provide meaningful descriptions of regulatory sequences. This was assessed in two ways. First, we used the TFcoop models to cluster mRNA promoters and searched for over-represented gene ontology (GO) terms in the inferred clusters. We randomly selected one model for each TF, and used the 106 selected models to score the 20,846 mRNA promoter sequences. Each promoter sequence was then described by a vector of length 106. We next ran a k-means algorithm to partition the promoters into 5 different clusters. For comparison, we ran the same procedure using two other ways to describe the promoter sequences: the classical PWM scores of the same 106 selected TFs (so promoters are also described by vectors of length 106), and the (di)nucleotide frequencies of the promoters (vector of length 12). We obtained three different clusterings of 5 clusters each. Then, we searched for over-represented GO terms in each clusters of the 3 clusterings. The rationale of this analysis was the following: a meaningful clustering should group together promoters of genes involved in the same biological functions, while a “random” partition should mix up promoters and prevent the observation of any over-representation of GO terms in clusters. Overall, the same 5 GO terms appeared to be over-represented in the different clusterings: defense response, immune system process, cell cycle, metabolic process, and developmental process. We noticed that the *p*-values obtained with the TFcoop scores were invariably better than the two others. To avoid any clustering bias, we repeated the k-means clusterings several times, with various numbers of clusters. Namely, for each approach we ran 3 clusterings for each number of clusters ranging between 3 and 10 (resulting in 24 different clusterings for each approach) and computed over-representation *p*-values for the 5 GO terms in each cluster. When the same GO term was enriched in several clusters of the same clustering, only the best *p*-value was kept. As shown in Fig. 7a, the TFcoop scores substantially and systematically outperform the other scoring functions, indicating that the classification obtained with this score is more accurate to functionally annotate promoters than the others. Implicitly, these results are also consistent with a model in which most biological processes are controlled by specific combinations of TFs.

Next, we used the TFcoop models to discriminate between mRNA promoters and enhancers. We randomly split the sets of promoters and enhancers in training and test sets, and learned a K-nearest neighbor (KNN) classifier for discriminating between promoter and enhancer sequences on the basis of scores of the TFcoop models learned on promoters. As above, we also used the classical PWM scores of the same 106 selected TFs and (di)nucleotide frequencies of the sequences. We resumed the procedure with a number of neighbors (K) varying between 1 and 20, and computed the number of errors obtained by each approach on the test set (Fig. 7b). Here again, TFcoop description outperforms other description methods, with an error rate around 2% for TFcoop vs. 15% and 25% for the other approaches. This result confirms the existence of DNA features distinguishing enhancers from mRNA promoters [21, 50] and identifies TF combinations as potent classifiers.

## Discussion

In this paper we proposed a method to identify TF combinations that can be predictive of the binding of a target TF. Our approach is based on a logistic model learned from ChIP-seq experiments on the target TF. Cross-validation study showed that the approach is effective and outperforms classical PWM based approaches on many TFs. It is important to note that TFcoop combinations do not necessarily reflect just cooperation, but also competition. For instance, a TF A competing with a TF B may be useful to predict the binding of B and would thus appear in the TF B model while A and B do not cooperate.

We distinguished two prediction problems associated with two situations, depending whether the aim is to predict binding in any promoter/enhancer or solely in expressed promoters/enhancers. For expressed promoters/enhancers, our experiments showed that the learned models have high TF specificity and quite low cell-type specificity. On the other hand, for the problem of expressed and not expressed promoters/enhancers binding, the learned models are less TF specific and more cell-type specific (especially for enhancers). These results are in accordance with a two-level model of gene regulation: (i) cell-type specific level that deposits specific chromatin marks on the genome, and (ii) non, or poorly, cell-type specific level that regulates TF binding in all DNA regions associated with appropriate marks.

An important property highlighted by our models is that rules governing TF combinations are very similar in the promoters of the three gene types analyzed (mRNA, pri-miRNA and lncRNA), but different between promoters and enhancers. Our results are in agreement with that of Andersson et al. [50], who showed that some motifs are enriched in enhancers (e.g. AP-1 or OCT4), while other are enriched in promoters (ELF1 or NRF1). We further confirmed these differences between promoter and enhancer sequences showing that scores produced by TFcoop models allow accurate classification between the two types of sequences. Our results thus argue for a prominent role of transcription factor binding as the fundamental determinant of regulatory activity able to distinguish enhancers and promoters [21]. Furthermore, as promoters and enhancers produce different RNA molecules [21, 50], our results also suggest that the production of enhancer RNAs (eRNAs) on one hand, and that of mRNAs, lncRNAs, and miRNAs on the other hand, requires a specific and distinct subset of TFs.

Our approach could be improved in several ways. A quite straightforward improvement would be to use the DNAshape score developed by Mathelier et al. [37] instead of the classical PWM score. This could improve TFcoop accuracy for several TFs, especially for TFs such as CTCF for which TFcoop does not outperform classical PWM scoring. More profoundly, one drawback of TFcoop is that the logistic model enables us to learn only a single TF combination for each target TF. However, we can imagine that certain TFs may be associated with two or more different TF combinations depending on the promoter/enhancer they bind. A solution for this would be to learn a discrimination function based on several logistic models instead of a single one.

## Conclusions

On the whole, studying TF combinations by the way of a TFBS prediction problem appears as a promising approach. We showed here that, despite its simplicity, the TFcoop method is accurate and allows identifying complex combinations on the basis of a single ChIP-seq experiment. We used it on ENCODE and FANTOM data and identified important properties of TF combinations in human. Specifically we showed that combinations governing TF binding on enhancers are more cell-type specific than that governing binding in promoters. Moreover, for a given TF and cell type, TF combinations are different between promoters and enhancers, but similar for promoters of mRNAs, lncRNAs and pri-miRNAs. Finally, analysis of the TFs cooperating with the different targets show over-representation of pioneer TFs and a clear preference for TFs with binding motif composition similar to that of the target.

## Methods

### Promoter, enhancer, long non-coding RNA and microRNA sequences

We predicted TF binding in both human promoters and enhancers. For promoters, sequences spanning ±500 bp around starts (i.e. most upstream TSS) of protein-coding genes, long non-coding RNAs and microRNAs were considered. Starts of coding and lncRNA genes were obtained from the hg19 FANTOM CAGE Associated Transcriptome (CAT) annotation [19, 49]. Starts of microRNA genes (primary microRNAs, pri-miRNAs) were from [20]. For enhancers, sequences spanning ±500 bp around the mid-positions of FANTOM-defined enhancers [50] were used. Lastly, our sequence datasets are composed of 20,845 protein coding genes, 1250 pri-microRNAs, 23,887 lncRNAs, and 38,553 enhancer sequences.

### Nucleotide and dinucleotide features

For each of these sequences, we computed nucleotide and dinucleotide relative frequencies as the occurrence number in the sequence divided by sequence length. Frequencies were computed in accordance with the rule of DNA reverse complement. For nucleotides, we computed the frequency of A/T and G/C. Similarly, frequencies of reverse complement dinucleotides (e.g. ApG and CpT) were computed together. This results in a total of 12 features (2 nucleotides and 10 dinucleotides).

### PWMs

We used vertebrate TF PFMs from JASPAR [23], including all existing versions of each PFM, resulting in a set of 638 PFMs with 119 alternative versions (i.e. 519 different TFs). We also used the non-redundant version of the JASPAR vertebrate database (519 PFMs) and the two alternative PFM libraries CisBP [25] and HOCOMOCO [24]. CisBP is a meta-library gathering PFMs from various sources, which contains up to 972 human PFMs (http://cisbp.ccbr.utoronto.ca). We collected, for each TF, all directly determined motifs indicated in TF_Information.txt of the Homo sapiens archive. To avoid redundancy, we selected only one model for each TF by arbitrarily selecting the longest PWM. Moreover we also excluded all TRANSFAC PWMs that are not publicly available (this reduces the set of TFs associated with a PFM to 625). Note that CisBP is built on JASPAR and HOCOMOCO 2014 versions. For HOCOMOCO, we used the human PCM v11 full collection of the core mononucleotide models (771 PCMs corresponding to 680 TFs). PCMs were converted into PFMs and PFMs were further transformed into PWMs as described in Wasserman and Sandelin [22]. PWM scores used by TFcoop for a given sequence were computed as described in [22], keeping the maximal score obtained in any position of the sequence. Namely, each PWM was used to scan the entire sequence and score each position, and the maximal score was used as potential predictive feature by TFcoop.

### ChIP-seq data

We collected ChIP-seq data from the ENCODE project [61] for human immortalized cell lines, tissues, and primary cells. Experiments were selected when the targeted TF were identified by a PWM in JASPAR. Thus we studied 409 ChIP-seq experiments for 106 distinct TFs and 41 different cell types. The most represented TF is CTCF with 69 experiments, while 88% of the experiments are designed from immortalized cell lines (mainly GM12878, HepG2 and K562). The detailed list of all used experiments is given in Supplementary materials. For each ChIP-seq experiment, regulatory sequences were classified as positive or negative for the corresponding ChIP targeted TF. We used Bedtools v2.25.0 [62] to detect intersection between ChIP-seq binding sites and regulatory sequences (both mapped to the hg19 genome). Each sequence that intersects at least one ChIP-seq binding region was classified as a positive sequence. The remaining sequences formed a negative set. The number of positive sequences varies greatly between experiments and sequence types. Mean and standard deviation numbers of positive sequences are respectively 2661(±1997) for mRNAs, 1699(±1151) for lncRNAs, 216(±176) for microRNAs, and 1516(±1214) for enhancers. For sake of comparison, we also used non-ENCODE ChIP-seq data collected from the Cistrome database [63] (http://cistrome.org). Note that Cistrome provides hg38 ChIP-seq peaks, not narrow peaks as provided by ENCODE. We collected data corresponding to GSM2224586 (ELF1), GSM1056931 (ETS1), GSM894076 (MAX), GSM1423725 (MYC), GSM1698353 (USF1), GSM1614036 (JUN), GSM2042914 (JUND), GSM1917774 (ATF3), GSM1708340 (YY1) and GSM1334010 (ZBTB33). The bed files were liftovered into hg19 coordinates using UCSC liftover tool.

### Expression data

To control the effect of expression in our analyses, we used ENCODE CAGE data restricted to 41 cell lines. The expression per cell line was calculated as the mean of the expression observed in all corresponding replicates. For microRNAs, we used the small RNA-seq ENCODE expression data collected for 3043 mature microRNAs in 37 cell lines (corresponding to 403 ChIP-seq experiments). The expression of microRNA genes (i.e. pri-microRNAs) was calculated as the sum of the expression of the corresponding mature microRNAs.

### Logistic model

We propose a logistic model to predict the regulatory sequences bound by a specific TF. Contrary to classical approaches, we not only consider the score of the PWM associated with the target TF, but also the scores of all other available PWMs. The main idea behind this is to unveil the TF interactions required for effective binding of the target TF. We also integrate in our model the nucleotide and dinucleotide compositions of the sequences, as the environment of TFBSs are thought to play major role in binding affinity [33, 34].

For each ChIP-Seq experiment, we learn different models to predict sequences bound by the target TF in four regulatory regions (promoters of mRNA, lncRNA and pri-miRNA, and enhancers). For a given experiment and regulatory region, our model aims to predict response variable *y*_*s*_ by the linear expression

$$\alpha + \sum_{m \in Motifs} \beta_{m} \times Score_{m,s} + \sum_{n \in Nucl} \beta_{n} \times Rate_{n,s} + \varepsilon_{s}, $$

where *y*_*s*_ is the Boolean response variable representing the TF binding on the given sequence *s* (*y*_*s*_=1 for TF binding, 0 otherwise); *Score*_*m*,*s*_ is the score of motif *m* on sequence *s*; *Rate*_*n*,*s*_ is the frequency of (di)nucleotide *n* in sequence *s*; *α* is a constant; *β*_*m*_ and *β*_*n*_ are the regression coefficients associated with motif *m* and (di)nucleotide *n*, respectively; and *ε*_*s*_ is the error associated with sequence *s*. *Motifs* and *Nucl* sets respectively contain 638 JASPAR PWMs and 12 (di)nucleotide frequencies.

To perform variable selection (i.e. identifying cooperating TFs), we used the LASSO regression minimising the prediction error within a constraint over *l*1-norm of *β* [48]. The weight of the LASSO penalty is chosen by cross-validation by minimising the prediction error with the R package *glmnet* [64] (see below).

### Cross-validations

TFcoop models were trained with the *cv*.*glmnet* function of the *glmnet* package, with options *nfolds*=10. This runs a 10-fold cross validation. In each validation loop, 90% of sequences are used to learn the *β* parameters and the remaining 10% are used to evaluate the predictive performance of the model. We set the option *keep*=*TRUE* to memorize the predictions achieved during cross-validation. These predictions were then systematically used in the AUC estimations to avoid over-fitting. There are two different situations here: when computing the AUC of a model trained on the same ChIP-seq data (for example in Fig. 1a) only the cross-validated predictions were used. However, when computing the AUC of a model trained on a different ChIP-seq data (for example in Fig. 1d), all test sequences do not belong to the training data (because all negative sequences were not used for training). In this case, we used the learned model to predict the outcome of the sequences that do not belong to the training data, and we used the prediction obtained during cross-validation by *cv*.*glmnet* for the other sequences.

### Alternative approaches

We compared the predictive accuracy of our model to three other approaches.

**Best hit approach** The traditional way to identify TF binding sites consists in scanning a sequence and scoring the corresponding PWM at each position. Positions with a score above a predefined threshold are considered as potential TFBS. A sequence is then considered as bound if it contains at least one potential TFBS.

**TRAP score** An alternative approach proposed by Roider et al. [54] is based on a biophysically inspired model that estimates the number of bound TF molecules for a given sequence. In this model, the whole sequence is considered to define a global affinity measure, which enables us to detect low affinity bindings. We use the R package *tRap* [64] to compute the affinity score of the 638 PWMs for all sequences. As proposed in [54], we use default values for the two parameters (*R*_0_(*width*), *λ*=0.7).

**DNA shape** In addition to PWMs, Mathelier et al. [37] considered 4 DNA shapes to increase binding site identification: helix twist, minor groove width, propeller twist, and DNA roll. The 2^*nd*^ order values of these DNA shapes are also used to capture dependencies between adjacent positions. Thus, each sequence is characterized by the best hit score for a given PWM plus the 1^*st*^ and 2^*nd*^ DNA shape order values at the best hit position. The approach based on gradient boosting classifier requires a first training step with foreground (bound) and background (unbound) sequences to learn classification rules. Then the classifier is applied to the set of test sequences. We used the same 10-fold cross-validation scheme that we used in our approach. We applied two modifications to speed-up the method, which was designed for smaller sequences. First, in the PWM optimization step of the training phase, we reduced the sequences to ±50 bp around the position with highest ChIP-Seq peak for positive sequences and to ±50 bp around a random position for negative sequences. Second, after this first step we also reduced sequences used to train and test the classifiers to ±50 bp around the position for which the (optimized) PWM gets the best score.

**DeepSEA** Zhou and Troyanskaya [45] proposed a deep learning approach for predicting the binding of chromatin proteins and histone marks from DNA sequences with single-nucleotide sensitivity. Their deep convolutional network takes 1000 bp genomic sequences as input and predicts the states associated with several chromatin marks in different tissues. We used the predictions provided by DeepSEA server (http://deepsea.princeton.edu/). Namely, coordinates of the analyzed promoter and enhancer sequences were provided to the server, and the predictions associated with each sequence were retrieved. Only the predictions related to the ChIP-seq data we used in our analyses were considered (i.e. 214 ChIP-seq data in total).

**Intersection between ChIP-seq experiments** We used the Jaccard index to assess the validity of the TF cooperations inferred by TFcoop or by the approach proposed in [13]. Namely, given two TFs A and B predicted to be cooperating in promoters (resp. enhancers), we identified the set of promoters (resp. enhancers) *X*_*A*_ with a ChIP-seq peak for TF A, and the set of promoters (resp. enhancers) *X*_*B*_ with a ChIP-seq peak for TF B, and measured the quantity

$$\text{Jaccard}(A,B) = \frac{X_{A} \cap X_{B}}{X_{A} \cup X_{B}}. $$

TFs that bind exactly the same sequences have a Jaccard index equal to 1, while TFs that bind exclusively different sequences have a Jaccard index equal to 0. For the method of Myšičková and Vingron, we used the TF pairs identified in the Additional file 1 of ref. [13]. For TFcoop, given a model predicting the presence of TF A, we enumerated all TFs B whose PWMs have been selected in model A. More precisely, the LASSO penalization allows us to rank the selected variables by order of importance (from the most to the less important variable). With this ranking, we used three cutoffs to distinguish three sets of PWMs: $S_{\lambda _{min}}$, $S_{\lambda _{1se}}$ and $S_{\lambda _{3se}}$, with $S_{\lambda _{3se}} \subset S_{\lambda _{1se}}\subset S_{\lambda _{min}}$. Set $S_{\lambda _{min}}$ contains all selected PWMs (which is by far smaller than the set of all possible PWMs), while sets $S_{\lambda _{1se}}$ and $S_{\lambda _{3se}}$ are restricted to the most important PWMs of $S_{\lambda _{min}}$. More precisely, *λ*_*min*_ is the penalization weight that gives minimum cross-validated error, while *λ*_1*se*_ (resp. *λ*_3*se*_) corresponds to penalization weights producing error within 1 (resp. 3) standard error of the minimum.

**Model clustering** We used the *kmeans* procedure implemented in R to classify the 2×409 models trained on promoters and enhancers. Each model was described by a Boolean vector describing the selected/non-selected variables (dinucleotides and PWMs). Different numbers of classes from 1 to 10 were tested. For each number, the kmeans was run 200 times and the best classification (according to the statistic optimized by the kmeans) was returned. To choose the “best” number of classes, we used a very simple procedure. We plotted the kmeans statistics vs. their corresponding class numbers and selected what can be considered as the best trade-off between modelling and complexity (see Additional file 1: Figure S10).

**GSEA analyzes** We used the GSEA program from the Broad Institute [55] to assess enrichment of specific annotations among the PWMs selected in our model. Different experiments have been done. In one experiment we ranked the PWMs by the number of models in which they appear (in promoters, and then in enhancers), and look for enrichment of certain JASPAR structural families (PWM annotations provided by JASPAR) or of pioneer factors (see below) in the PWMs at the top of the list. In other experiments, PWMs were ranked by their difference of utilization between models learned in the expression controlled experiments and non-expression controlled experiment (see Additional file 1: Table S1) or between promoter models and enhancer models (Additional file 1: Table S2) and we looked for enrichment of certain JASPAR structural families in the top PWMs of these lists.

**Pioneer factors** We used the classification of [9] to distinguish pioneers, settlers and migrants TFs. ’Pioneer’ TFs occupy previously closed chromatin and, once bound, allow other TFs to bind nearby. ’Settler’ designate TFs whose binding is predominantly dependent on the openness of chromatin at their motifs. ’Migrants’ bind only sporadically even when chromatin at their motifs is open.

## Additional file

Additional file 1 **Figure S1.** Comparison of the accuracy of the different approaches on the 409 experiments in the non expression-controlled challenge for promoters. (a) TRAP vs. Best hit, (b) DNA shape vs. Best hit, (c) TFcoop vs. Best hit, (d) TFcoop vs. DNA shape. **Figure S2.** ROC curves obtained on mRNA promoters for the 409 ChIP-seq experiments (non expression-controlled challenge). **Figure S3.** Link between the number of training sequences (x-axis) and model AUCs (y-axis). **Figure S4.** Comparison of AUCs achieved when using nucleotide and dinucleotide frequencies only (x-axis) and when using nucleotide, di-, tri-, and quadri-nucleotide frequencies (y-axis). **Figure S5.** Comparison of AUCs achieved with the JASPAR (complete), JASPAR (non-redundant), CisBP and HOCOMOCO databases of PWMs. **Figure S6.** Comparison of AUCs achieved on ENCODE and non-ENCODE data. Each column corresponds to a TFcoop model learned on a specific ENCODE ChIP-seq experiment. Black points correspond to AUC achieved when using these models on other ENCODE ChIP-seq data targeting the same TF, while red triangles correspond to the AUC achieved when using these models on a non-ENCODE ChIP-seq targeting the same TF. Globally, AUCs achieved on non-ENCODE data are in the range of the AUCs achieved on ENCODE data. **Figure S7.** Enrichment of three different PWM classes in the selected PWMs of promoter (up) and enhancer (down) models. For these analyses, PWMs were ranked according to the number of times they have been selected in promoter and enhancer models, and the GSEA method was applied to identify over-represented PWM classes among most used PWMs. **Figure S8.** Mean rank of the selected dinucleotides in promoter models according to the dinucleotide composition of the corresponding target PWM. For each model, the 16 dinucleotide variables were ordered according to their frequency in the target PWM. Then, the rank of each dinucleotide was averaged for all models. High mean rank thus indicates that, when selected, the dinucleotide was also frequent in the target PWM. **Figure S9.** Enrichment of pioneer factors among selected PWMs for promoters (a) and enhancers (b). For these analyses, PWMs were ranked according to the number of times they have been selected in promoter and enhancer models, and the GSEA method has been applied to compute the enrichment of pioneers among most used PWMs. **Figure S10.** (Up): Heatmap of the selected variables in the 409 logistic models learned on the mRNA promoters in the expression-controlled challenge. Each column corresponds to one of the logistic model, while the rows represent the variables used in the models (PWM affinity scores and mono- and di-nucleotide frequencies). Models (columns) have been partitioned in 5 different classes (represented by different colors on the top line) by a k-means algorithm. The number of classes 5 was empirically chosen because it shows good trade-off between modelling and complexity. (Down): Trade-off between modelling and complexity. This figure reports the average distance (y-axis) between points in the same class, according to the number of classes of the classification (x-axis). Until 5 classes, we can observe substantial decrease of the average distance between points, while after 5 classes the decrease is slighter and almost linear. **Figure S11.** The 30 most common variables in the five classes of models represented in Additional file 1: Figure 10. Each bar represents the proportion of models (in the class) which use the considered variable. Dark bars represent TFs classified as “pioneers factors” in the reference [9], while pale bars correspond to TF classified as “settler” or “migrant” in the same publication. Plain bars correspond to non-classified TFs as well as to mono- or di-nucleotides. **Figure S12.** AT rate distributions of selected PWMs in mRNA promoter models (with *β*>0). For each cluster we keep one model per target PWM to avoid bias due to overrepresentation of some PWMs. As cluster 4 is only composed of CTCF models, the distribution associated with this cluster is represented by a vertical segment on the x-axis. **Figure S13.** Distribution of methylation binding influence in selected PWMs of mRNA promoter models. We kept one model for each target PWM to avoid bias due to over-representation of the same PWM in certain classes. In grey is represented the distribution of all PWMs associated with a methylation class originally defined in reference [51] (190 over 520 non redundant PWMs). “Little” designates TFs recognizing CpG-containing sequences, but methylation of the CpG has little effect on binding. “MethylMinus” refers to TFs, which do not bind to, or more weakly to, methylated versions of their recognition sequences. Conversely, TFs that prefer to bind to methylated sequences over the corresponding unmethylated sequence belong to the “MethylPlus” class. see [51] for further details. **Figure S14.** Distribution of the number of mRNA and miRNA promoters overlapping a ChIP-seq peak in the 409 ChIP-seq experiments. **Figure S15.** Promoter models are interchangeable. Left: AUC comparaison of models learned and applied on lncRNAs and of models learned on mRNAs and applied on lncRNAs. Right: AUC comparaison of models learned and applied on pri-miRNAs and of models learned on mRNAs and applied on pri-miRNAs. **Figure S16.** Comparison of the accuracy of the different approaches on the 409 experiments in the non expression-controlled challenge for enhancers. (a) TRAP vs. Best hit, (b) DNA shape vs. Best hit, (c) TFcoop vs. Best hit, (d) TFcoop vs. DNA shape. **Figure S17.** Distribution of Gini coefficients computed for 53,220 gene promoters and 65,423 FANTOM5 enhancers on 1827 and 1897 samples, respectively. Gini coeficient is a measure of statistical dispersion which can be used to measure gene ubiquity: value 0 represents genes expressed in all samples, while value 1 represents genes expressed in only one sample. **Figure S18.** Heatmap of the selected variables in the 409 logistic models learned on the mRNA enhancers in the expression-controlled challenge. Each column corresponds to one of the logistic model, while the rows represent the variables used in the models (PWM affinity scores and mono- and di-nucleotide frequencies). Models (columns) have been partitioned in 6 different classes (represented by different colors on the top line) by a k-means algorithm. **Figure S19.** The 30 most common variables in the six classes of models represented in Additional file 1: Figure S18. Each bar represents the proportion of models (in the class) which use the considered variable. Dark bars represent TFs classified as “pioneers factors” in the reference [9], while pale bars correspond to TF classified as “settler” or “migrant” in the same publication. Plain bars corresponds to non-classified TFs as well as to mono- or di-nucleotides. **Figure S20.** AT rate distributions of selected PWMs in enhancer models (with *β*>0). For each cluster we keep one model per target PWM to avoid bias due to overrepresentation of some PWMs. **Figure S21.** Dotplot of the AUCs computed on mRNA promoter and on enhancers for the same ChIP-seq experiment. **Table S1.** Variables that are more selected in the non-controlled models than in the corresponding expression-controlled models in promoters (left) and enhancers (right). # ¬contr.: number of non controlled models that involve each variable. # contr.: number of corresponding expression-controlled models that also involve the variable. *P*-values were computed by hypergeometric tests. **Table S2.** Variables that are differentially selected in promoters and enhancers. (left) variables more selected in promoter models than in enhancers. (right) variables more selected in enhancer models than in promoters. # promo: number of promoter models involving this variable. # enhancer: number of enhancer models involving this variable. *P*-values were computed by chi2 test. (PDF 4198 kb)
