# Supplementary material for: Probing transcription factor combinatorics in different promoter classes and in enhancers
Source: BMC Genomics. 2019 Feb 1;20:103. doi: 10.1186/s12864-018-5408-0 (PMC6359851; doi:10.1186/s12864-018-5408-0)
Supplement: Supplementary file 1 — Figure S1. Comparison of the accuracy of the different approaches on the 409 experiments in the non expression-controlled challenge for promoters. (a) TRAP vs. Best hit, (b) DNA shape vs. Best hit, (c) TFcoop vs. Best hit, (d) TFcoop vs. DNA shape. Figure S2. ROC curves obtained on mRNA promoters for the 409 ChIP-seq experiments (non expression-controlled challenge). Figure S3. Link between the number of training sequences (x-axis) and model AUCs (y-axis). Figure S4. Comparison of AUCs achieved when using nucleotide and dinucleotide frequencies only (x-axis) and when using nucleotide, di-, tri-, and quadri-nucleotide frequencies (y-axis). Figure S5. Comparison of AUCs achieved with the JASPAR (complete), JASPAR (non-redundant), CisBP and HOCOMOCO databases of PWMs. Figure S6. Comparison of AUCs achieved on ENCODE and non-ENCODE data. Each column corresponds to a TFcoop model learned on a specific ENCODE ChIP-seq experiment. Black points correspond to AUC achieved when using these models on other ENCODE ChIP-seq data targeting the same TF, while red triangles correspond to the AUC achieved when using these models on a non-ENCODE ChIP-seq targeting the same TF. Globally, AUCs achieved on non-ENCODE data are in the range of the AUCs achieved on ENCODE data. Figure S7. Enrichment of three different PWM classes in the selected PWMs of promoter (up) and enhancer (down) models. For these analyses, PWMs were ranked according to the number of times they have been selected in promoter and enhancer models, and the GSEA method was applied to identify over-represented PWM classes among most used PWMs. Figure S8. Mean rank of the selected dinucleotides in promoter models according to the dinucleotide composition of the corresponding target PWM. For each model, the 16 dinucleotide variables were ordered according to their frequency in the target PWM. Then, the rank of each dinucleotide was averaged for all models. High mean rank thus indicates that, when selected, the dinucleotide [file 12864_2018_5408_MOESM1_ESM.pdf]

Probing transcription factor combinatorics in different promoter classes and in enhancers

Jimmy Vandel [1,2], Océane Cassan [1,2], Sophie Lèbre [1,3,†], Charles-Henri Lecellier [1,4,†,*], Laurent Bréhélin [1,2,†*]

[1] Institut de Biologie Computationnelle, Montpellier, France , [2] LIRMM, CNRS, Univ. Montpellier, Montpellier, France , [3] IMAG, CNRS, Univ. Montpellier, Montpellier, France , [4] IGMM, CNRS, Univ. Montpellier, Montpellier, France , [†]These authors contributed equally to this work

REFERENCES

1. Richard I. Sherwood, Tatsunori Hashimoto, Charles W. O'Donnell, Sophia Lewis, Amira A. Barkal, John Peter van Hoff, Vivek Karun, Tommi Jaakkola, and David K. Gifford. Discovery of directional and nondirectional pioneer transcription factors by modeling DNase profile magnitude and shape. *Nature Biotechnology*, 32(2):171–178, February 2014.
2. Y. Yin, E. Morgunova, A. Jolma, E. Kaasinen, B. Sahu, S. Khund-Sayeed, P. K. Das, T. Kivioja, K. Dave, F. Zhong, K. R. Nitta, M. Taipale, A. Popov, P. A. Ginno, S. Domcke, J. Yan, D. Schubeler, C. Vinson, and J. Taipale. Impact of cytosine methylation on DNA binding specificities of human transcription factors. *Science*, 356(6337), May 2017.
3. Jian Zhou and Olga G. Troyanskaya. Predicting effects of noncoding variants with deep learning-based sequence model. *Nature Methods*, 12(10):931–934, October 2015.

*To whom correspondence should be addressed. Emails: brehelin@lirmm.fr and charles.lecellier@igmm.cnrs.fr

© The Author(s)

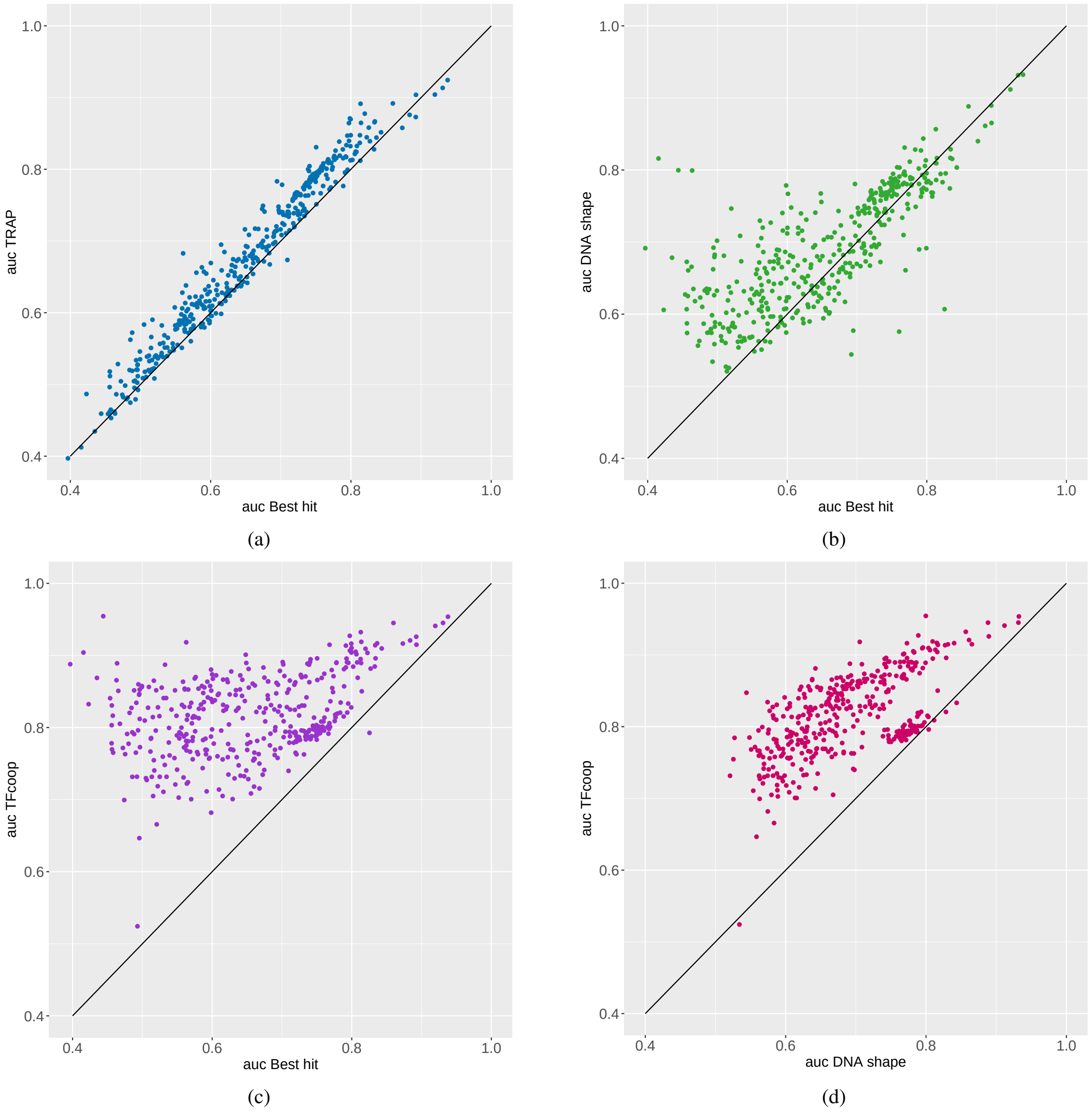

(a) (b) (c) (d)

Figure S1. Comparison of the accuracy of the different approaches on the 409 experiments in the non expression-controlled challenge for promoters. (a) TRAP *vs.* Best hit, (b) DNA shape *vs.* Best hit, (c) TFcoop *vs.* Best hit, (d) TFcoop *vs.* DNA shape

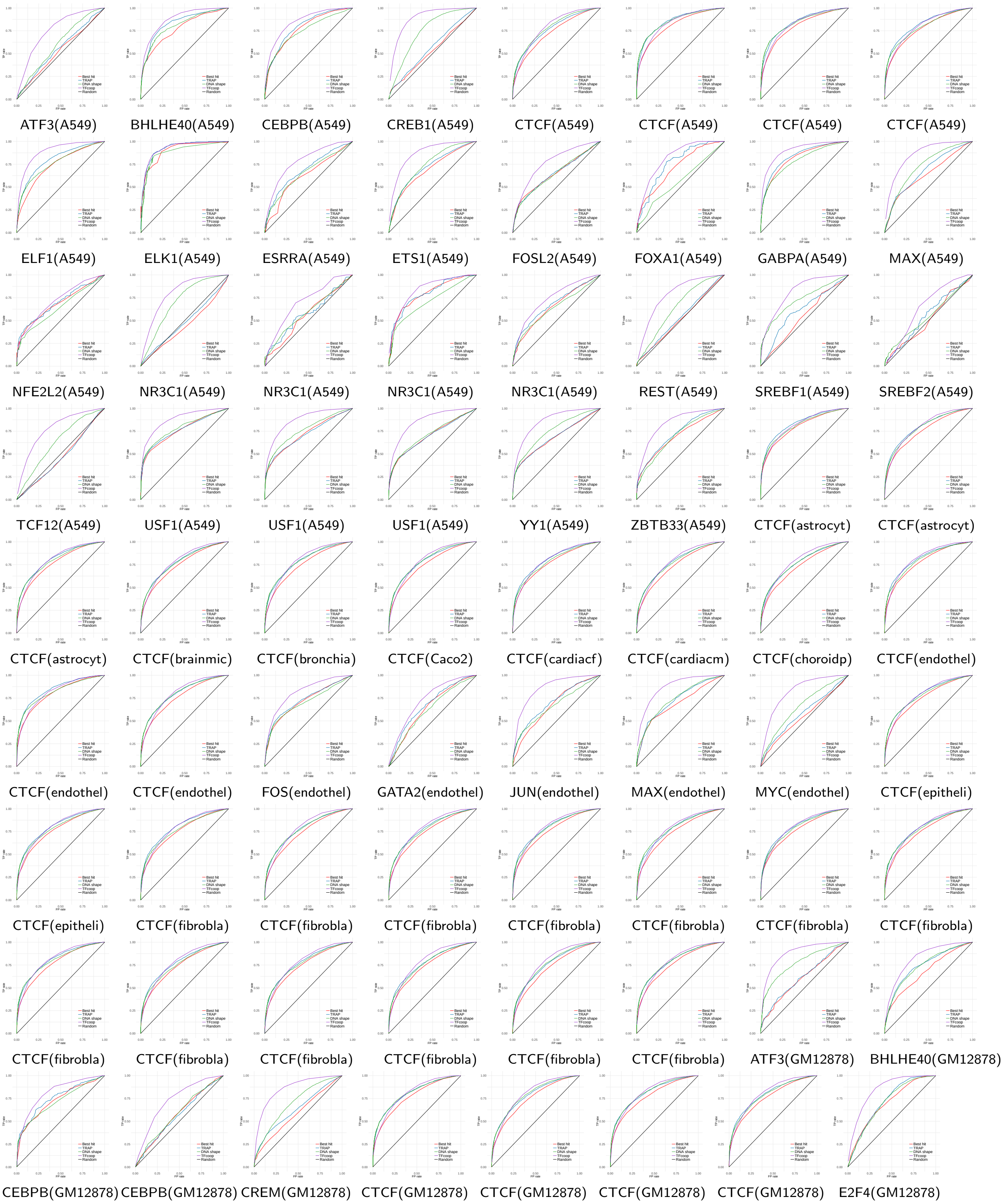

Figure S2. ROC curves obtained on mRNA promoters for the 409 ChIP-seq experiments (non expression-controlled challenge).

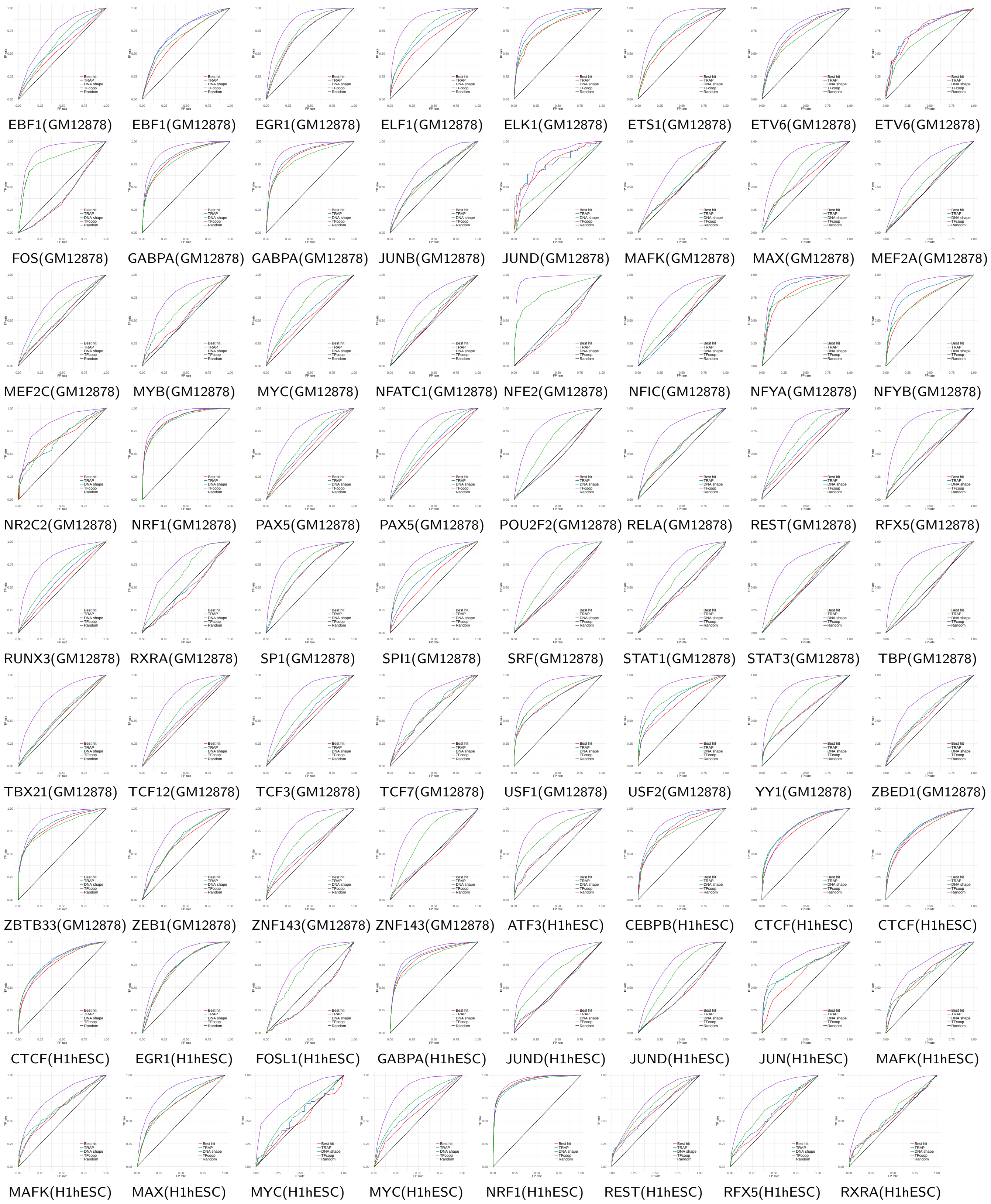

Figure S2. continue…

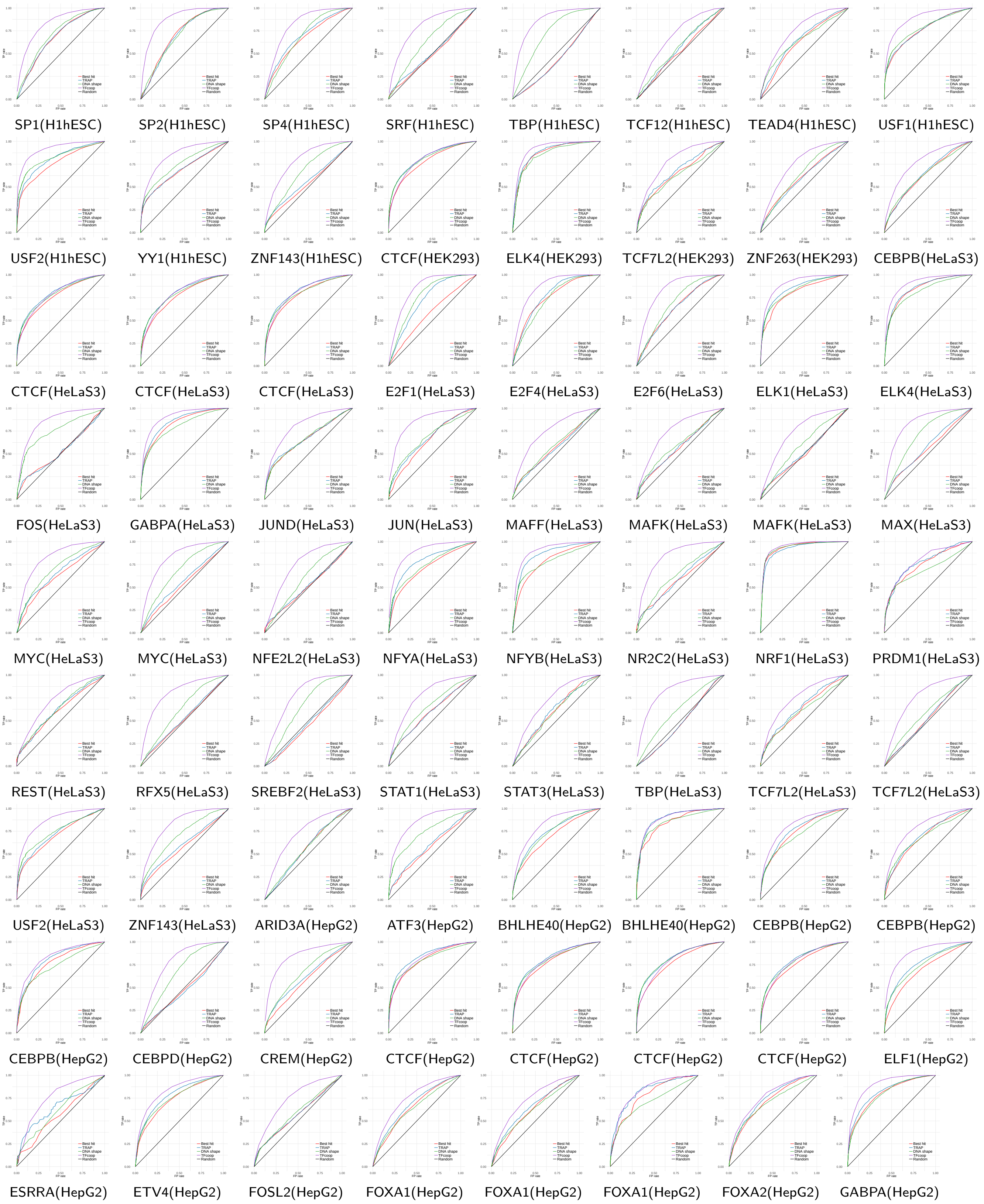

Figure S2. continue…

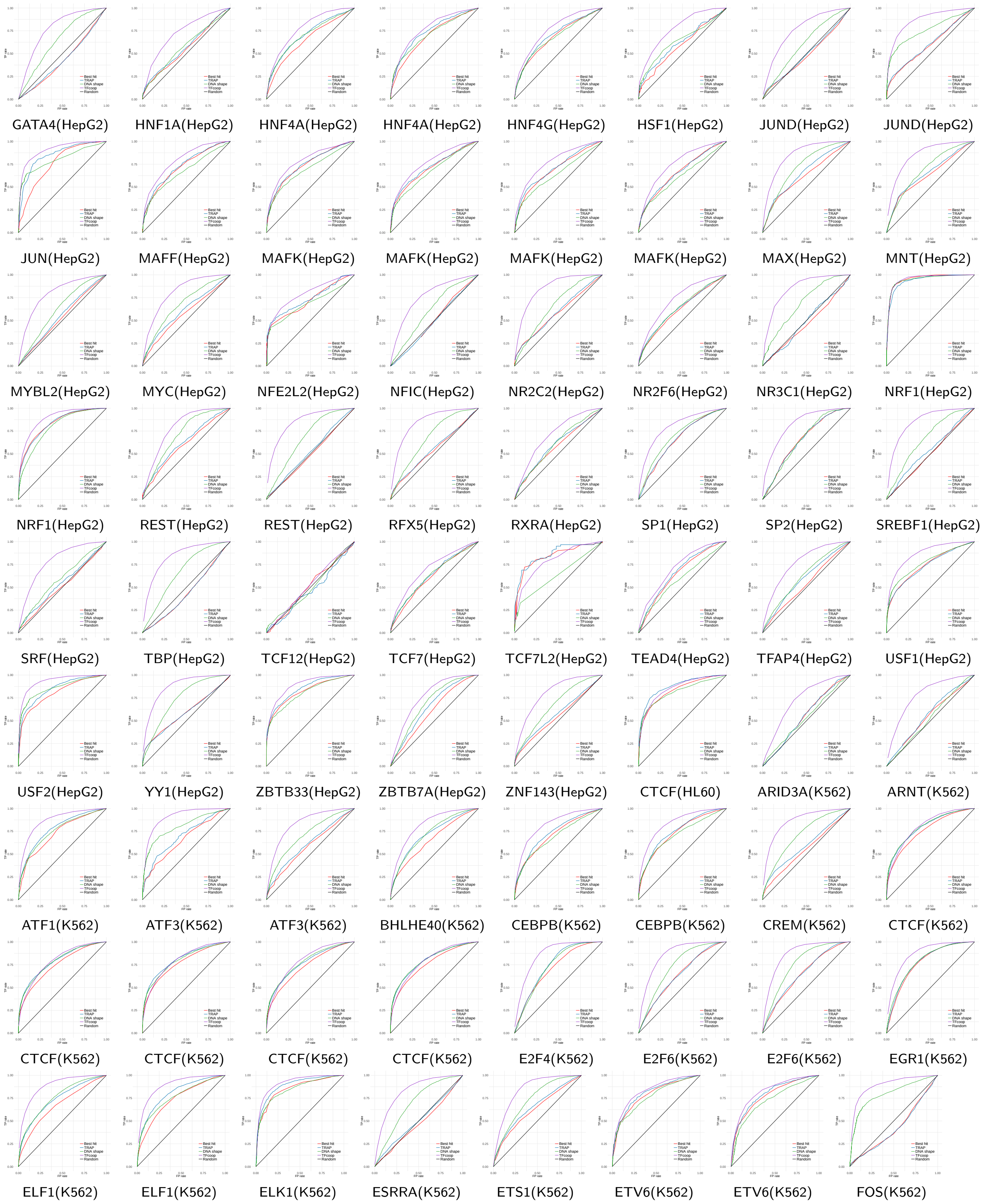

Figure S2. continue…

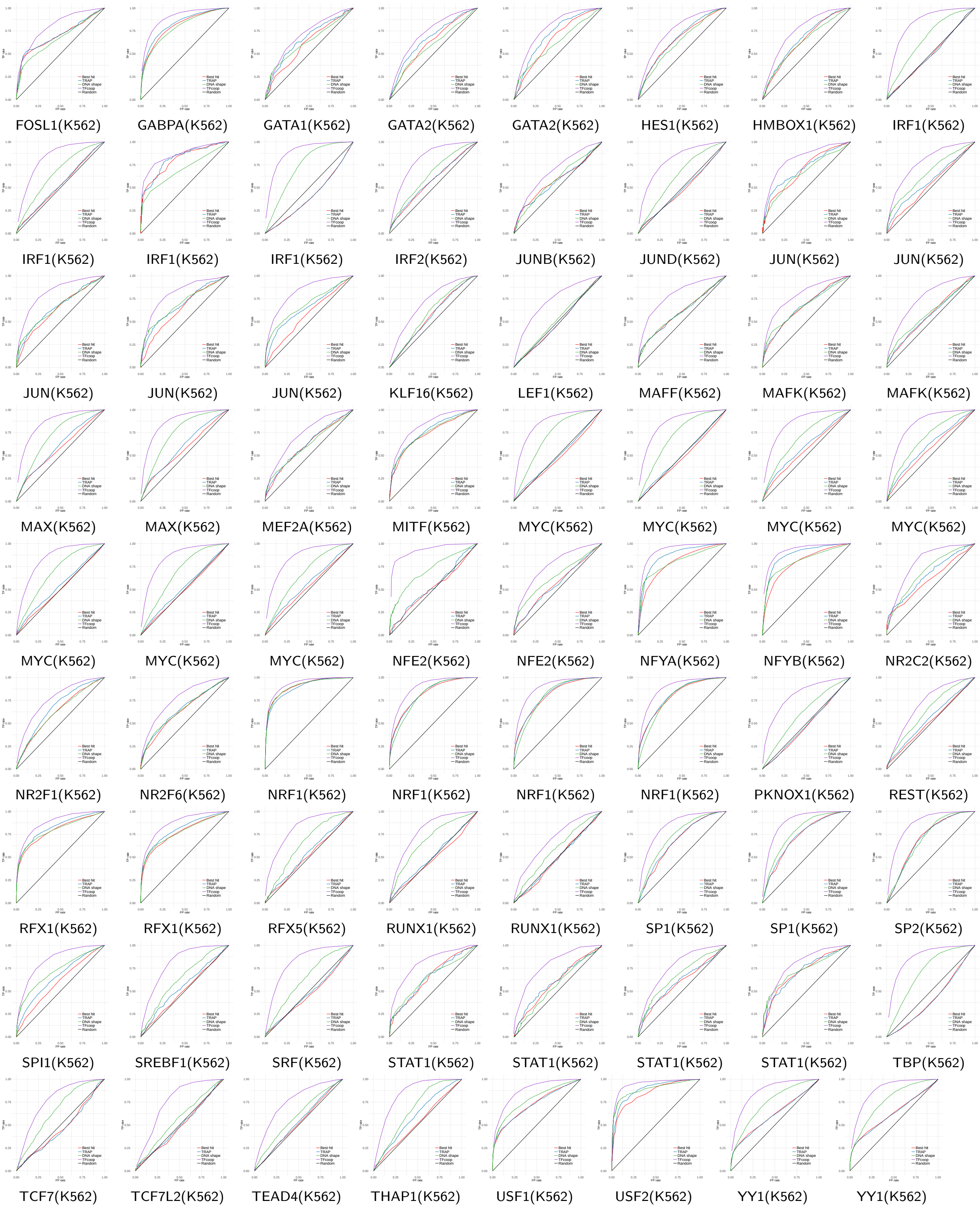

Figure S2. continue…

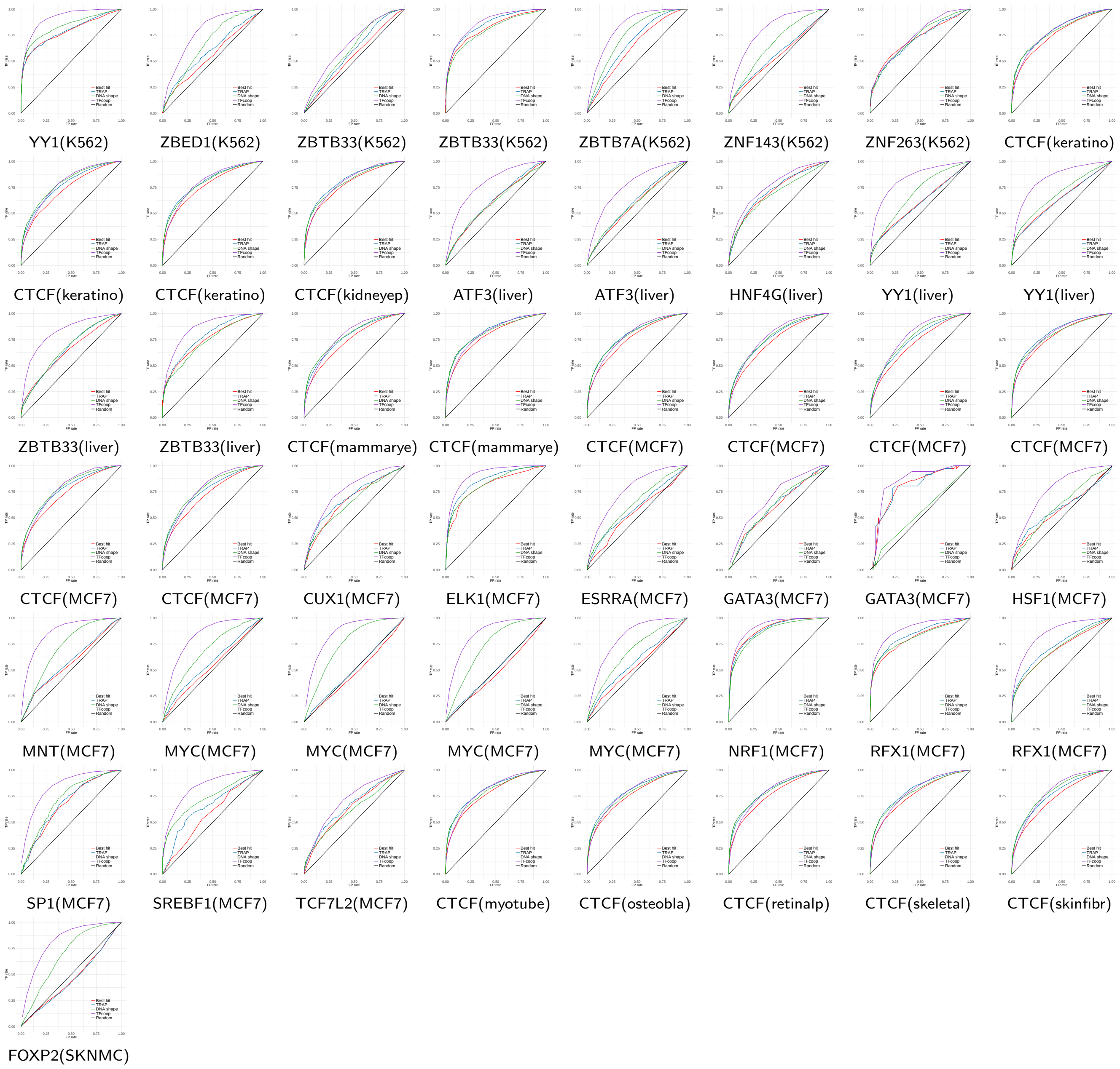

Figure S2. continue…

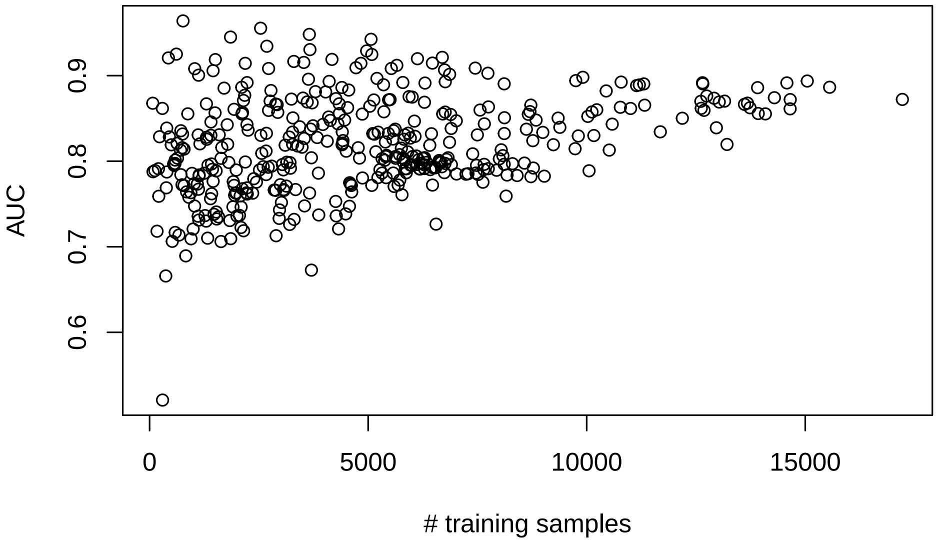

Figure S3. Link between the number of training sequences (x-axis) and model AUCs (y-axis).

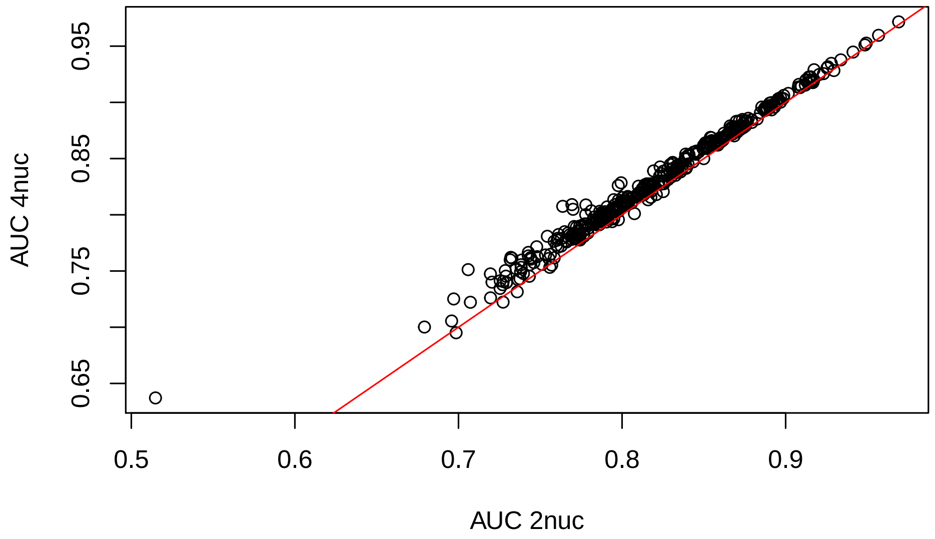

Figure S4. Comparison of AUCs achieved when using nucleotide and dinucleotide frequencies only (x-axis) and when using nucleotide, di-, tri-, and quadri-nucleotide frequencies (y-axis).

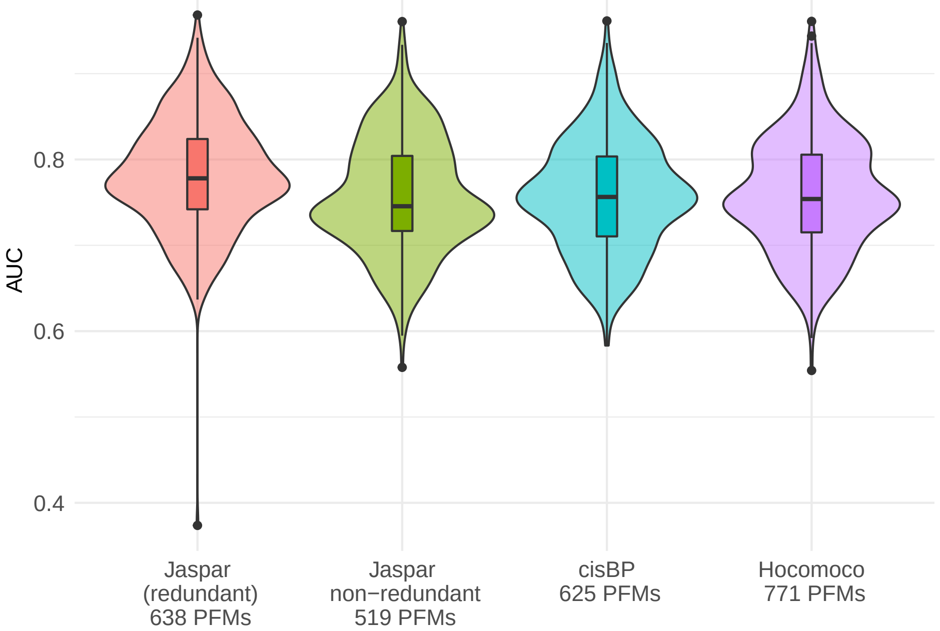

Figure S5. Comparison of AUCs achieved with the JASPAR (complete), JASPAR (non-redundant), CisBP and HOCOMOCO databases of PWMs.

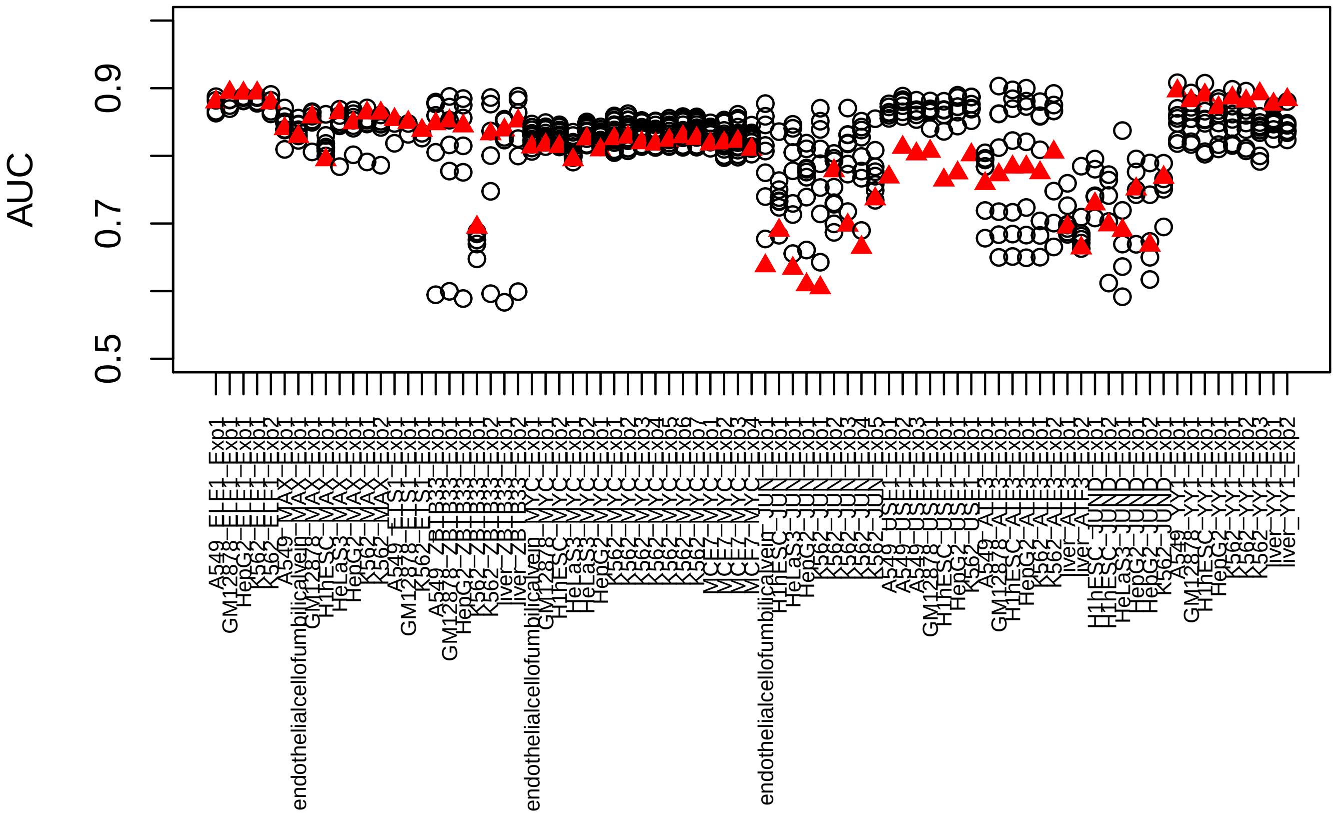

Figure S6. Comparison of AUCs achieved on ENCODE and non-ENCODE data. Each column corresponds to a TFcoop model learned on a specific ENCODE ChIP-seq experiment. Black points correspond to AUC achieved when using these models on other ENCODE ChIP-seq data targeting the same TF, while red triangles correspond to the AUC achieved when using these models on a non-ENCODE ChIP-seq targeting the same TF. Globally, AUCs achieved on non-ENCODE data are in the range of the AUCs achieved on ENCODE data.

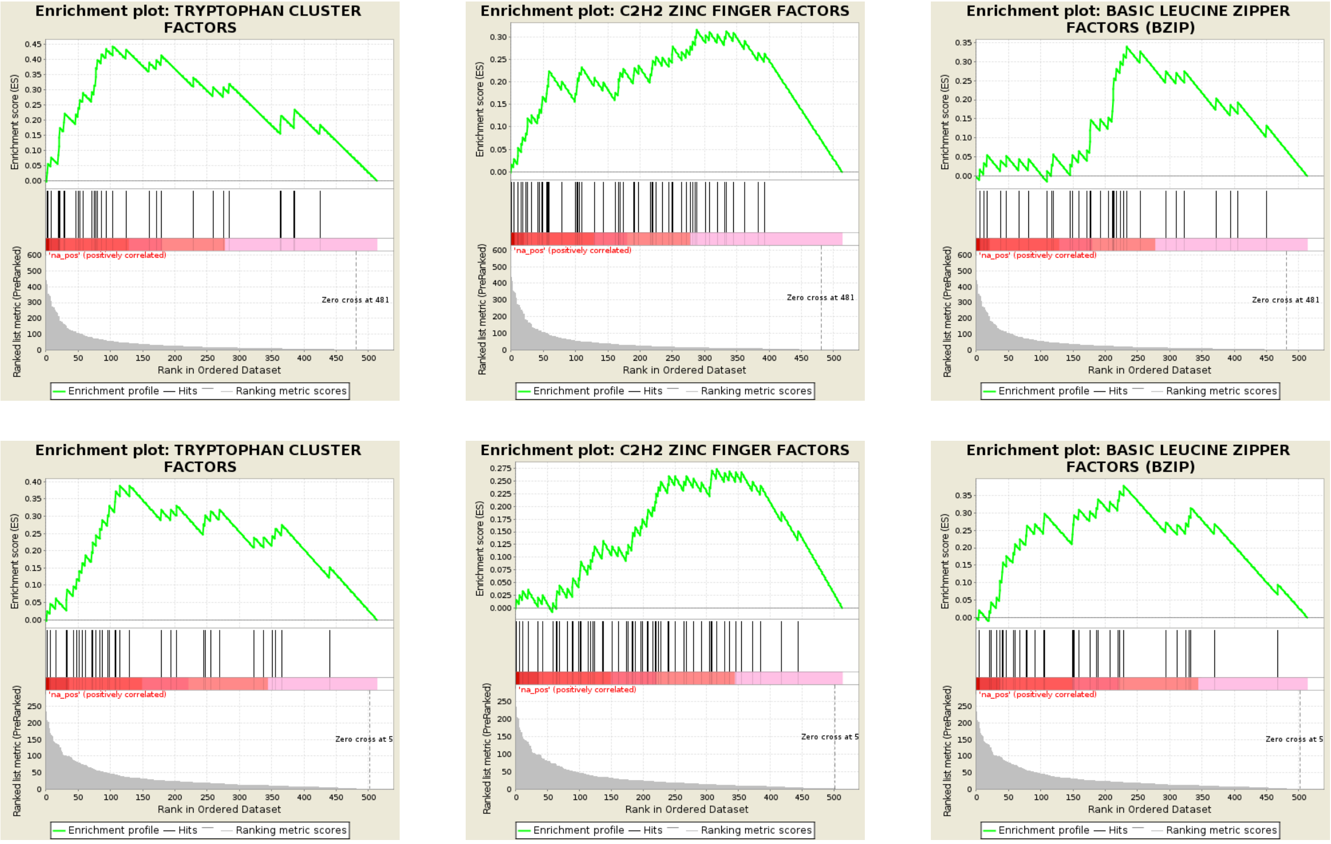

Figure S7. Enrichment of three different PWM classes in the selected PWMs of promoter (up) and enhancer (down) models. For these analyses, PWMs were ranked according to the number of times they have been selected in promoter and enhancer models, and the GSEA method was applied to identify over-represented PWM classes among most used PWMs.

PWM/nuc.	# ¬contr.	# contr.	p-value	PWM/nuc.	# ¬contr.	# contr.	p-value
ZFX	175	41	3.14e-17	CpG	228	36	1.05e-08
FOXD3	17	0	1.47e-06	SP1	173	24	2.11e-08
NR3C1	37	6	1.67e-06	TpC	72	7	6.77e-06
EBF1	68	18	2.29e-06	ApT	149	25	1.44e-05
T	15	0	7.16e-06	NFIA	27	0	2.71e-05
TFAP2C(VAR.2)	22	2	1.03e-05	KLF12	39	3	2.97e-04
PPARG::RXRA	28	4	1.22e-05	DMRT3	20	0	4.15e-04
PAX9	57	15	1.36e-05	KLF16	66	9	4.59e-04
FOXP2	14	0	1.58e-05	VDR	19	0	6.12e-04
HLF	27	4	2.31e-05	NR3C1	51	6	6.67e-04
OTX1	17	1	3.16e-05	NHLH1	64	9	7.57e-04
SOX17	39	9	6.07e-05	YY1	133	26	7.63e-04
FOXA2	46	12	8.14e-05	GABPA	61	9	1.57e-03
FOXH1	25	4	8.22e-05	SMAD2::D3::D4	16	0	1.97e-03
TP53	38	9	1.04e-04	TFAP2B	16	0	1.97e-03
ELF4	15	1	1.36e-04	E2F4	50	7	2.81e-03
PAX5	52	15	1.51e-04	EN2	15	0	2.91e-03
TCF3	47	13	1.66e-04	TWIST2	21	1	3.09e-03
FOXC2	11	0	1.69e-04	ZNF143	45	6	3.28e-03
HOXD13	11	0	1.69e-04	ETV5	82	15	3.49e-03
ZBTB7B	29	6	2.02e-04	MECOM	26	2	3.49e-03
IRF9	72	24	2.19e-04	FOSL2	65	11	4.26e-03
TBP	26	5	2.44e-04	HINFP	77	14	4.30e-03
IRF7	31	7	2.86e-04	TP53	20	1	4.37e-03
ESR2	36	9	2.98e-04	GSC	20	1	4.37e-03
SMAD2::D3::D4	17	2	3.22e-04	SPDEF	30	3	4.66e-03
VDR	17	2	3.22e-04	FOXH1	25	2	4.79e-03
MEIS2	28	6	3.57e-04	PAX4	13	0	6.34e-03
EHF	70	24	4.83e-04	SOX5	13	0	6.34e-03
NKX3-2	35	9	4.99e-04	HOXA5	13	0	6.34e-03
ZEB1	54	17	5.05e-04	TBR1	13	0	6.34e-03
ESRRB	22	4	5.22e-04	CREB3L1	13	0	6.34e-03
STAT6	13	1	5.79e-04	CDX1	13	0	6.34e-03
GLI2	32	8	6.51e-04	SPZ1	24	2	6.56e-03
XBP1	24	5	7.81e-04	ZNF410	24	2	6.56e-03
SMAD3	9	0	8.19e-04	SP4	74	14	7.78e-03

Table S1. Variables that are more selected in the non-controlled models than in the corresponding expression-controlled models in promoters (lefts) and enhancers (right). #¬contr.: number of non controlled models that involve each variable. # contr.: number of corresponding expression-controlled models thatalsoinvolvethevariable.P-valueswerecomputedbyhypergeometrictests.

PWM/nuc.	# promo.	# enhancer	p-value	PWM/nuc.	# promo.	# enhancer	p-value
E2F1_1	290	78	2.03e-57	GpC	0	129	9.84e-36
ATF1_1	259	51	6.77e-56	JUNB_1	6	98	4.02e-22
HINFP_1	230	28	7.09e-56	TEAD4_1	11	93	7.44e-18
YY2_1	247	41	7.37e-56	NFE2L2_2	27	120	1.38e-17
CpA	291	88	5.41e-53	JUN(VAR.2)_1	20	99	4.21e-15
GABPA_2	238	43	1.37e-50	FOXA1_1	13	82	6.17e-14
ZBTB33_1	209	22	1.53e-50	NFE2_1	23	97	2.46e-13
NRF1_1	286	88	2.20e-50	SP1_2	16	83	8.14e-13
YY1_2	243	49	1.60e-49	MAF::NFE2_1	34	110	2.34e-12
ETV5_1	274	82	2.88e-47	GATA1_2	8	65	4.35e-12
ZNF143_2	170	9	1.08e-43	FOXA2_2	19	85	4.93e-12
TCFL5_1	232	58	3.27e-40	JUND_1	17	81	6.77e-12
ApC	234	66	2.93e-37	GATA5_1	30	102	6.87e-12
GABPA_1	160	19	7.40e-34	CEBPA_3	3	50	4.95e-11
ELK1_1	275	117	6.15e-33	ETS1_3	4	51	1.04e-10
ZBED1_1	146	16	6.51e-31	BACH1::MAFK_1	51	126	1.24e-10
ELK4_2	150	27	2.71e-26	YY1_1	22	84	1.26e-10
CEBPG_1	125	13	5.33e-26	FOXA1_2	9	61	1.32e-10
ZNF143_1	159	36	8.15e-25	FOS_1	28	93	1.58e-10
MYB_1	174	47	1.23e-24	GATA1::TAL1_1	30	96	1.61e-10
ZFX_1	113	10	4.07e-24	TEAD1_1	25	88	1.90e-10
SPI1_1	120	14	6.28e-24	BATF::JUN_1	22	83	2.14e-10
AHR::ARNT_1	202	71	7.21e-24	ERG_2	15	71	2.41e-10
SP2_1	174	55	1.98e-21	FOXD2_1	6	53	3.89e-10
CENPB_1	151	39	3.94e-21	RUNX1_2	8	57	4.08e-10
GMEB2_1	118	19	1.00e-20	ETS1_2	12	64	5.79e-10
ETV6_1	160	48	4.30e-20	FOS::JUN_1	55	125	2.42e-09
GATA2_1	130	30	5.06e-19	TEAD3_1	23	79	3.82e-09
E2F1_3	116	22	9.98e-19	IRF1_2	6	48	6.33e-09
NFYA_1	188	74	1.09e-18	KLF16_1	16	66	8.46e-09
E2F4_1	157	50	1.65e-18	CEBPE_1	11	57	9.29e-09
RREB1_1	93	10	2.15e-18	SPIC_1	4	43	9.53e-09
HINFP_2	150	49	5.33e-17	FOXO1_1	7	47	3.30e-08
SP4_1	180	74	1.53e-16	HLF_2	5	42	5.42e-08
ELF3_1	69	4	2.48e-15	JDP2_1	20	68	8.40e-08
KLF14_1	99	21	1.14e-14	EGR1_2	21	69	1.11e-07
E2F1_2	118	36	1.46e-13	FOXO3_1	8	46	1.59e-07
BARHL2_1	88	19	9.18e-13	FOSL2_1	19	65	1.66e-07
TFAP2A_1	169	78	1.09e-12	HNF4A_2	20	66	2.20e-07
BHLHE41_1	80	15	1.63e-12	RARA::RXRA_1	14	56	2.40e-07
ELF1_2	84	18	3.33e-12	EGR1_1	26	75	2.43e-07
DUX_1	67	10	1.34e-11	GATA1_1	23	70	3.02e-07
EHF_2	54	4	1.84e-11	STAT3_1	19	63	4.33e-07
CREB1_2	103	32	2.00e-11	ZIC3_1	6	40	4.81e-07
FOXC1_1	68	11	2.24e-11	HNF1B_2	14	54	6.45e-07
ARNT::HIF1A_1	98	29	2.57e-11	CEBPB_1	7	40	1.35e-06
KLF12_1	112	39	3.69e-11	SPI1_4	18	59	1.35e-06
STAT3_2	74	15	4.69e-11	FOSL1_1	37	86	1.87e-06
HIC2_1	93	27	7.09e-11	SPI1_3	30	76	2.09e-06
TCF4_1	78	18	9.02e-11	ETV4_1	8	41	2.13e-06

Table S2. Variables that are differentially selected in promoters and enhancers. (left) variables more selected in promoter models than in enhancers. (right) variables more selected in enhancer models than in promoters. # promo: number of promoter models involving this variable. # enhancer: number ofenhancermodelsinvolvingthisvariable.P-valueswerecomputedbychi2test.

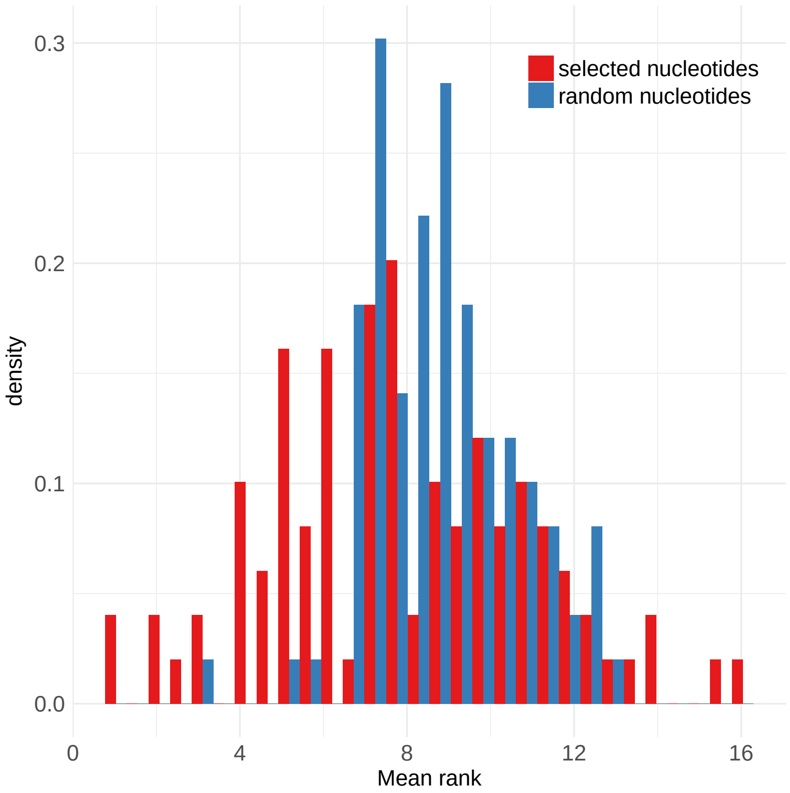

Figure S8. Mean rank of the selected dinucleotides in promoter models according to the dinucleotide composition of the corresponding target PWM. For each model, the 16 dinucleotide variables were ordered according to their frequency in the target PWM. Then, the rank of each dinucleotide was averagedfor allmodels.Highmeanrankthusindicatesthat,whenselected,thedinucleotidewasalsofrequentinthetargetPWM.

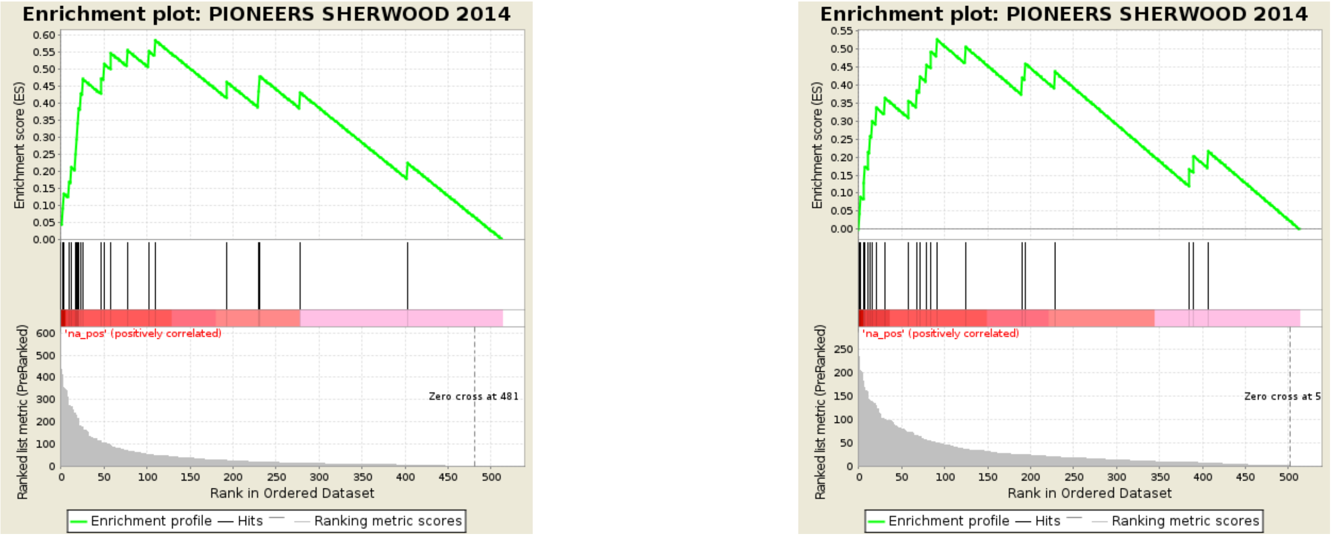

(a) (b)

Figure S9. Enrichment of pioneer factors among selected PWMs for promoters (a) and enhancers (b). For these analyses, PWMs were ranked according to the number of times they have been selected in promoter and enhancer models, and the GSEA method has been applied to compute the enrichment of pioneersamongmostusedPWMs.

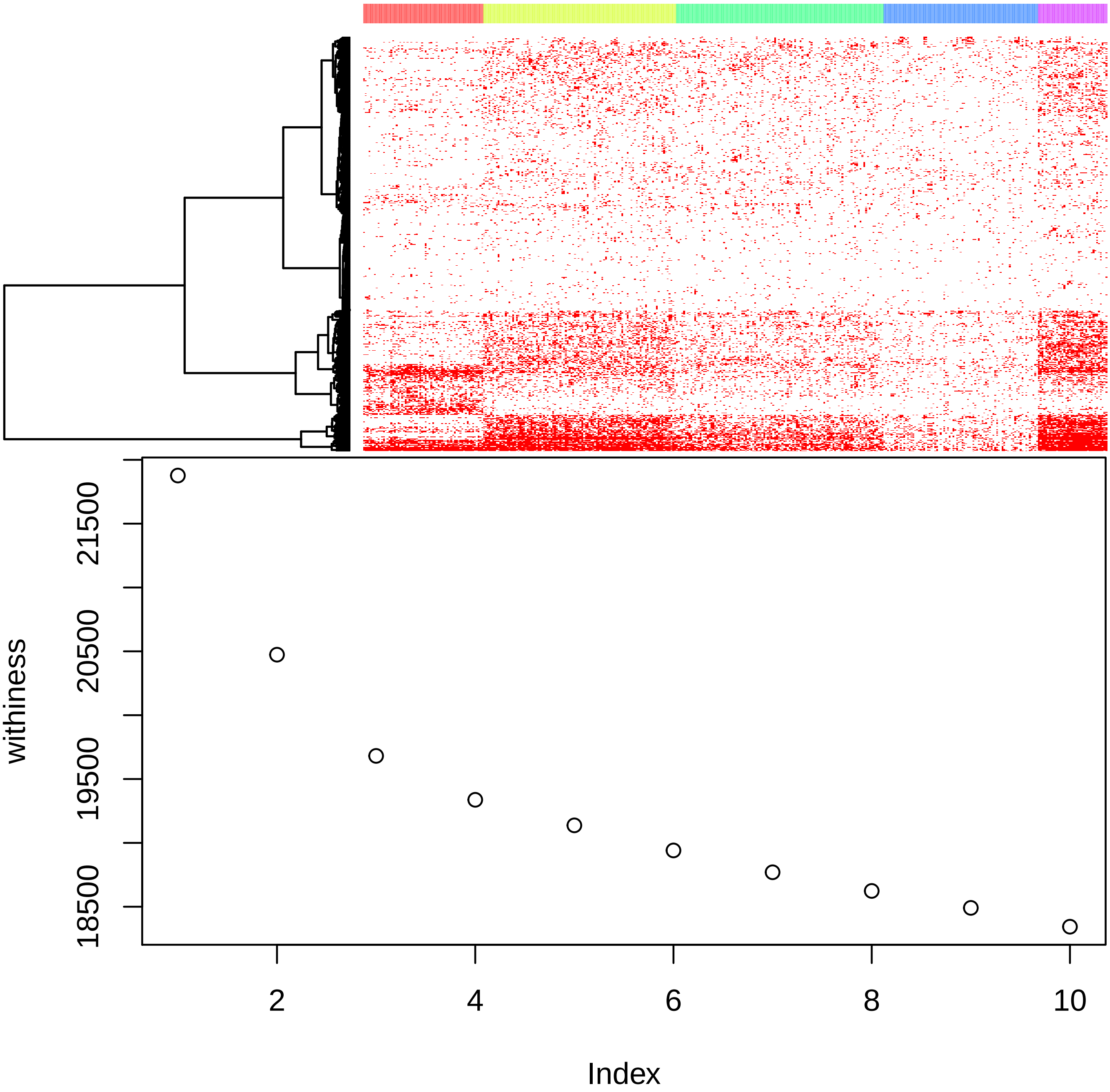

Figure S10. (Up): Heatmap of the selected variables in the 409 logistic models learned on the mRNA promoters in the expression-controlled challenge. Each column corresponds to one of the logistic model, while the rows represent the variables used in the models (PWM affinity scores and mono- and di-nucleotide frequencies). Models (columns) have been partitioned in 5 different classes (represented by different colors on the top line) by a k-means algorithm. The number of classes 5 was empirically chosen because it shows good trade-off between modelling and complexity. (Down): Trade-off between modelling and complexity. This figures reports the average distance (y-axis) between points in the same class, according to the number of classes of the classification (x-axis). Until 5 classes, we can observe substantial decrease of the average distance between points, while after 5 classes the decrease is slighterandalmostlinear.

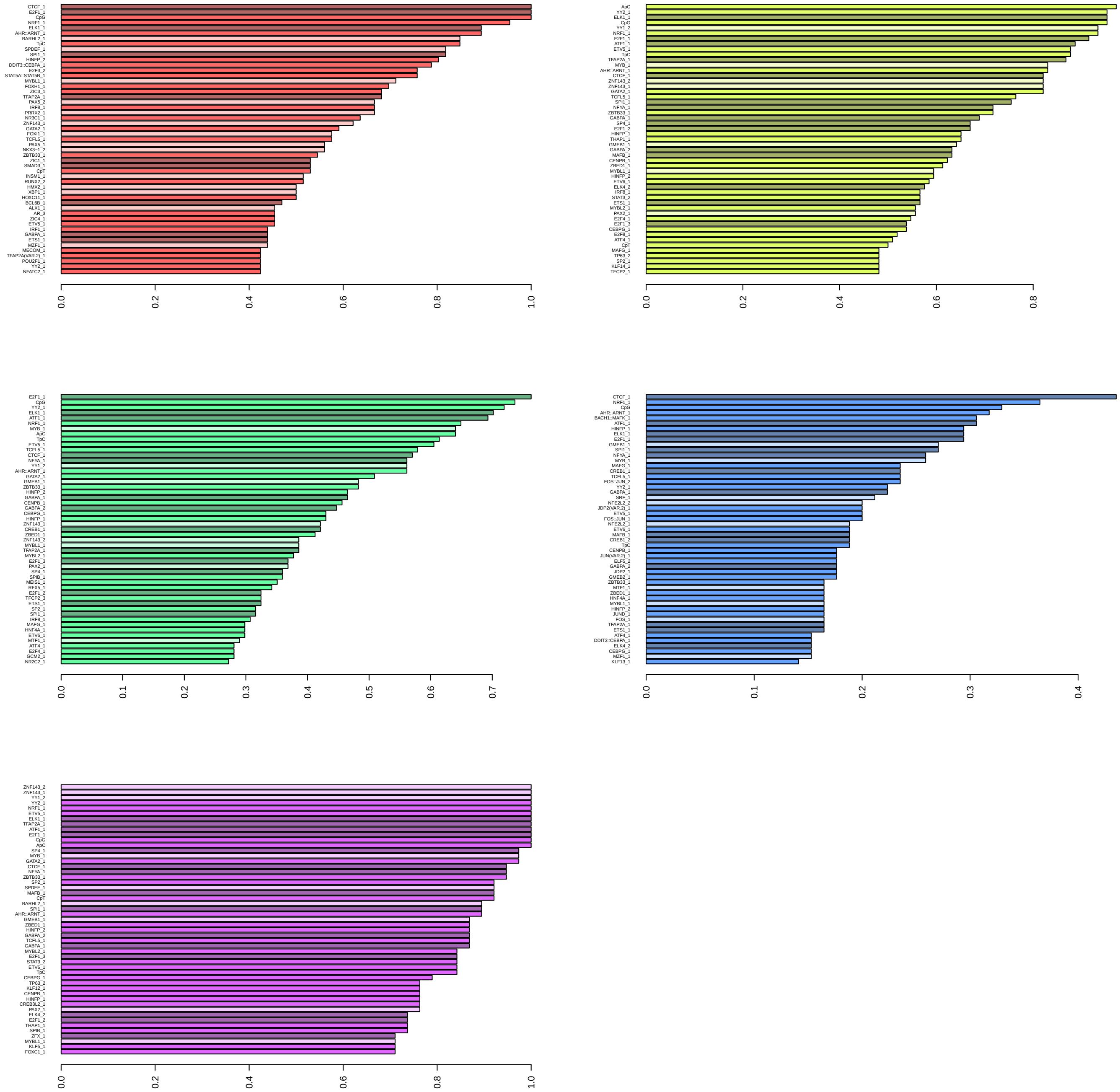

Figure S11. The 30 most common variables in the five classes of models represented in Supp. Figure 10. Each bar represents the proportion of models (in the class) which use the considered variable. Dark bars represent TFs classified as "pioneers factors" in the reference (1), while pale bars correspond toTF classifiedas"settler"or"migrant"inthesamepublication.Plainbarscorrespondstonon-classifiedTFsaswellastomono-ordi-nucleotides.

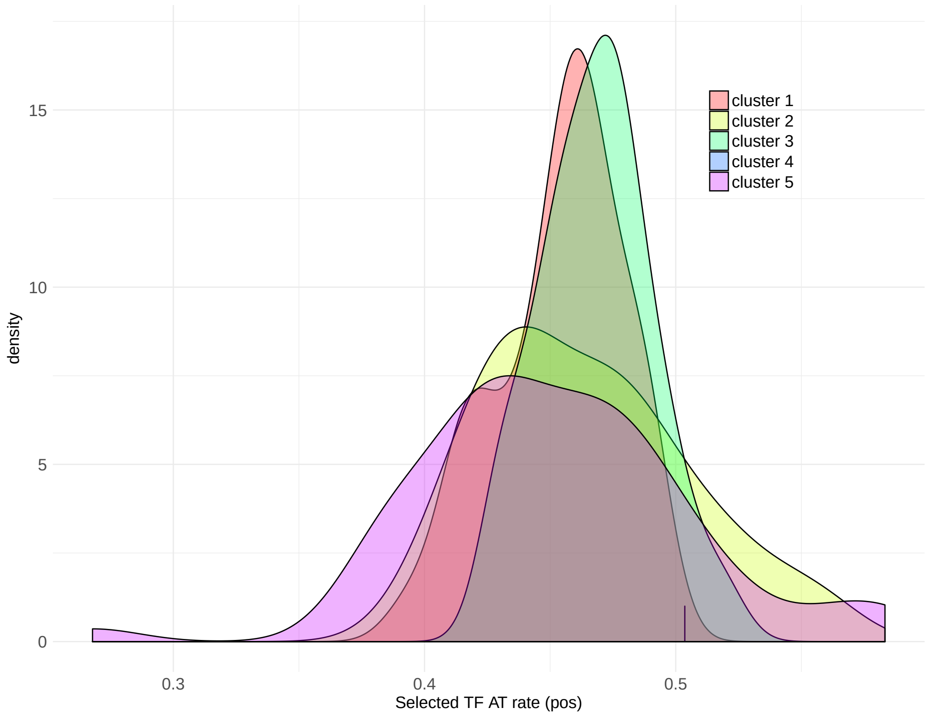

Figure S12. AT rate distributions of selected PWMs in mRNA promoter models (with $\beta > 0$). For each cluster we keep one model per target PWM to avoid bias due to overrepresentation of some PWMs. As cluster 4 is only composed of CTCF models, the distribution associated with this cluster is representedbyaverticalsegmentonthex-axis.

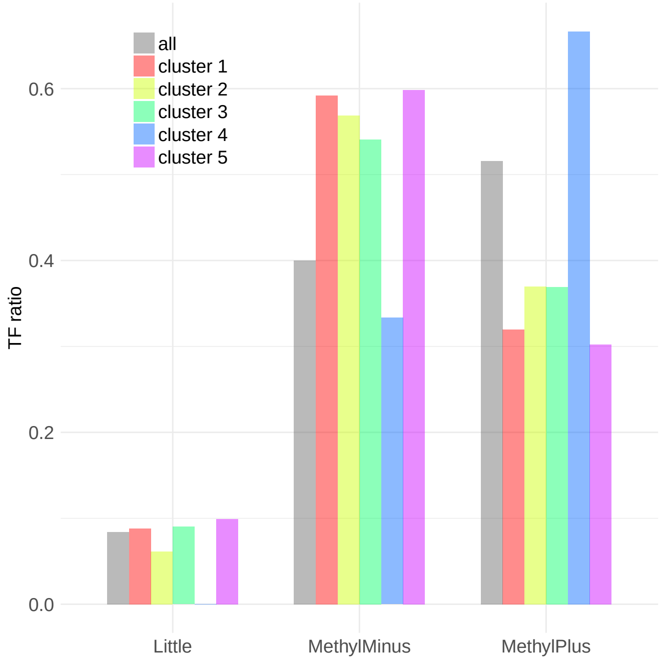

Figure S13. Distribution of methylation binding influence in selected PWMs of mRNA promoter models. We kept one model for each target PWM to avoid bias due to over-representation of the same PWM in certain classes. In grey is represented the distribution of all PWMs associated with a methylation class originally defined in reference (2) (190 over 520 non redundant PWMs). "Little" designates TFs recognizing CpG-containing sequences, but methylation of the CpG has little effect on binding. "MethylMinus" refers to TFs, which do not bind to, or more weakly to, methylated versions of their recognition sequences. Conversely, TFs that prefer to bind to methylated sequences over the corresponding unmethylated sequence belong to the MethylPlus"class.see(2)forfurtherdetails.

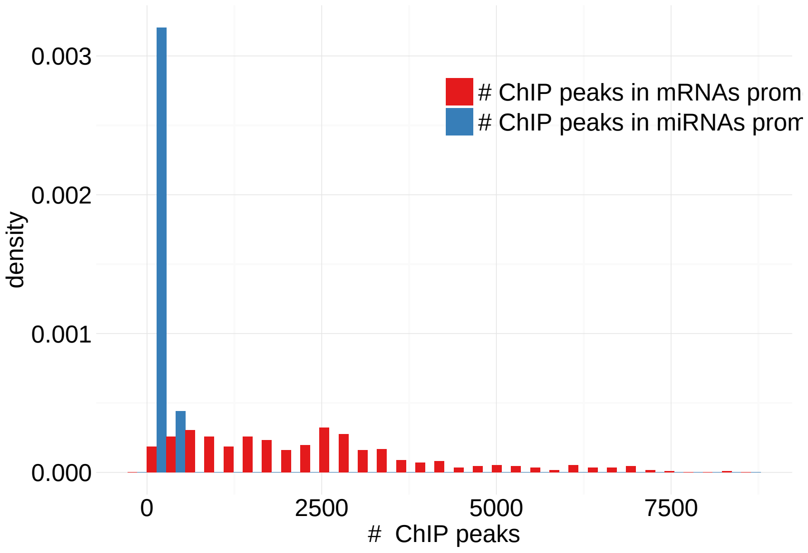

Figure S14. Distribution of the number of mRNA and miRNA promoters overlapping a ChIP-seq peak in the 409 ChIP-seq experiments.

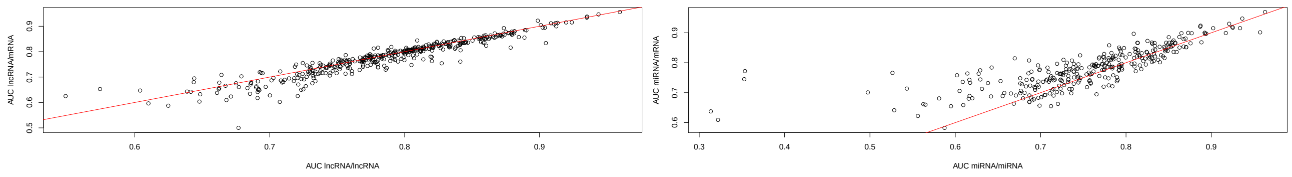

Figure S15. Promoter models are interchangeable. Left: AUC comparaison of models learned and applied on lncRNAs and of models learned on mRNAs and applied on lncRNAs. Right: AUC comparaison of models learned and applied on pri-miRNAs and of models learned on mRNAs and appliedon pri-miRNAs.

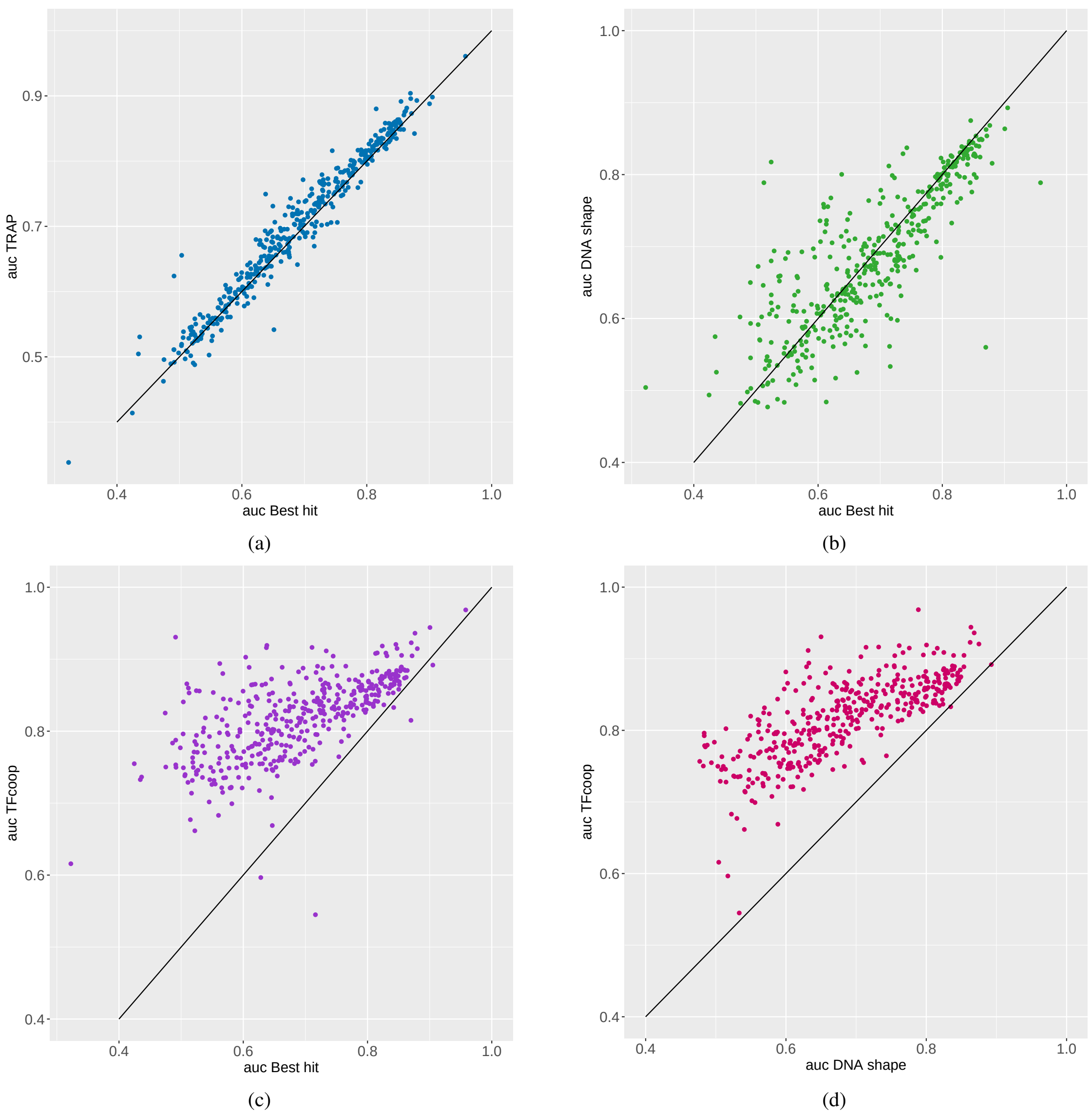

Figure S16. Comparison of the accuracy of the different approaches on the 409 experiments in the non expression-controlled challenge for enhancers. (a) TRAP *vs.* Best hit, (b) DNA shape *vs.* Best hit, (c) TFcoop *vs.* Best hit, (d) TFcoop *vs.* DNA shape

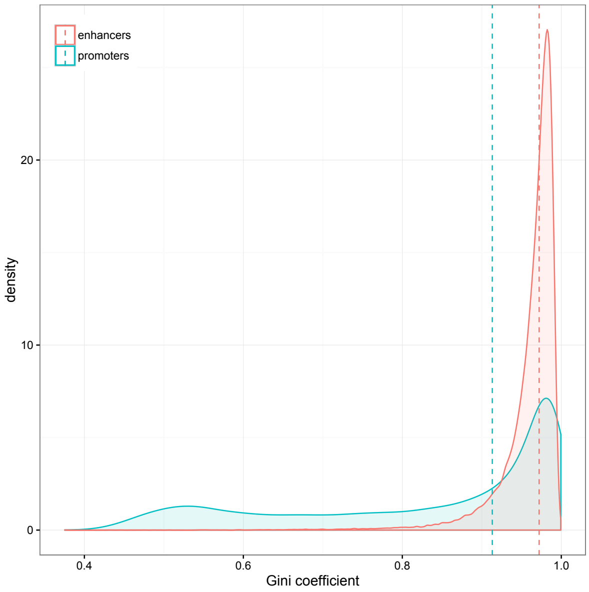

Figure S17. Distribution of Gini coefficients computed for 53,220 gene promoters and 65,423 FANTOM5 enhancers on 1,827 and 1,897 samples, respectively. Expression data were obtained at http://fantom.gsc.riken.jp/5/data/. Gini coeficient is a measure of statistical dispersion which can beusedto measuregeneubiquity:value0representsgenesexpressedinallsamples,whilevalue1representsgenesexpressedinonlyonesample.

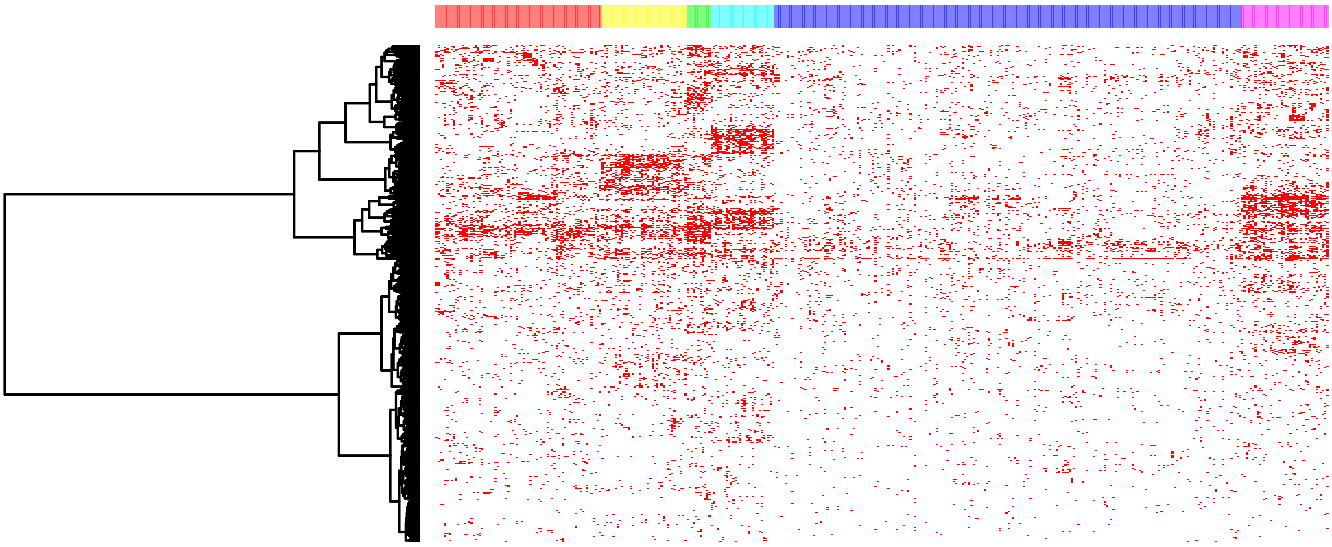

Figure S18. Heatmap of the selected variables in the 409 logistic models learned on the mRNA enhancers in the expression-controlled challenge. Each column corresponds to one of the logistic model, while the rows represent the variables used in the models (PWM affinity scores and mono- and di-nucleotide frequencies). Models(columns)havebeenpartitionedin6differentclasses(representedbydifferentcolorsonthetopline)byak-meansalgorithm.

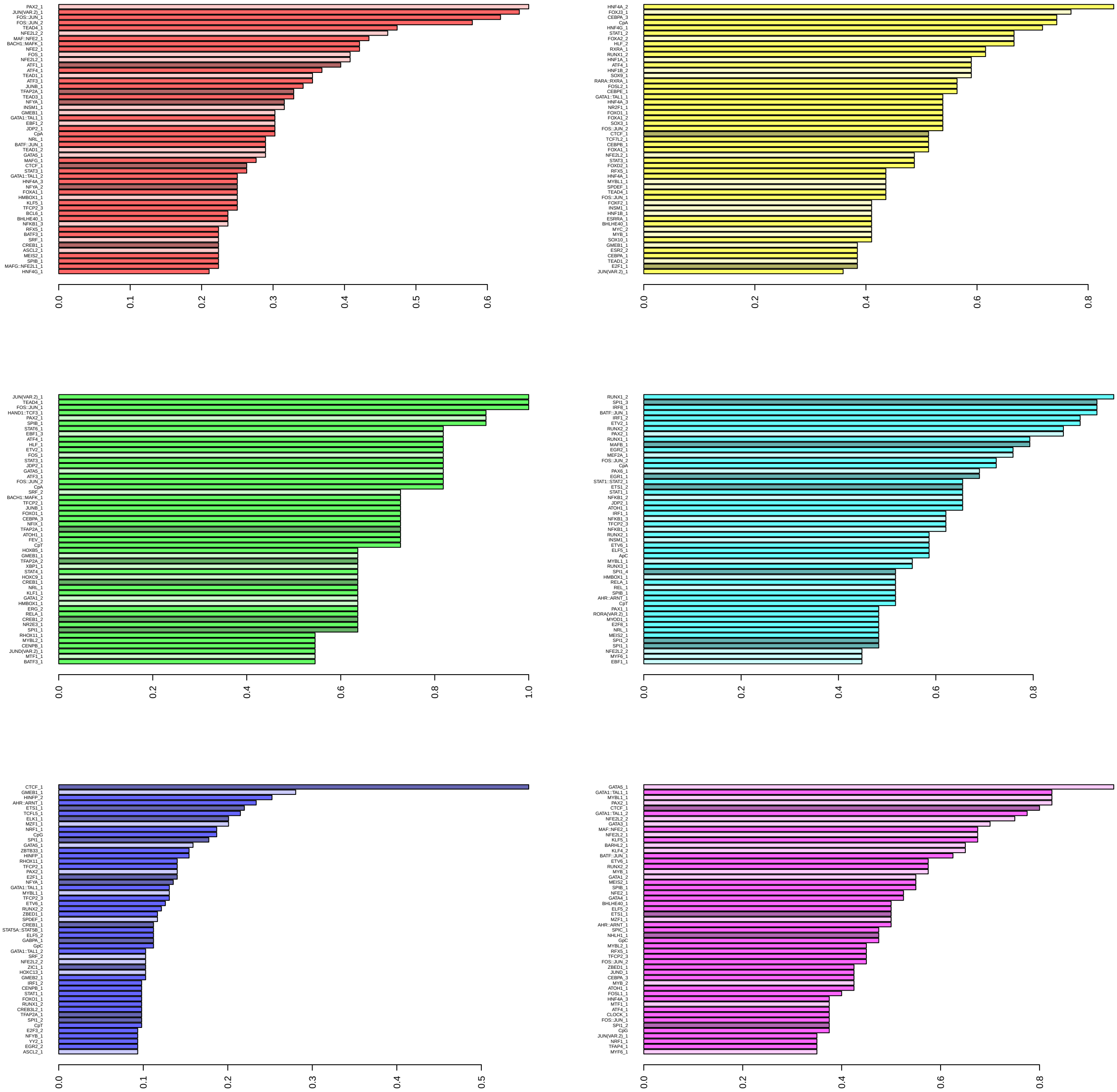

Figure S19. The 30 most common variables in the six classes of models represented in Supp. Figure 18. Each bar represents the proportion of models (in the class) which use the considered variable. Dark bars represent TFs classified as “pioneers factors” in the reference (1), while pale bars correspond toTF classifiedas“settler”or“migrant”inthesamepublication.Plainbarscorrespondstonon-classifiedTFsaswellastomono-ordi-nucleotides.

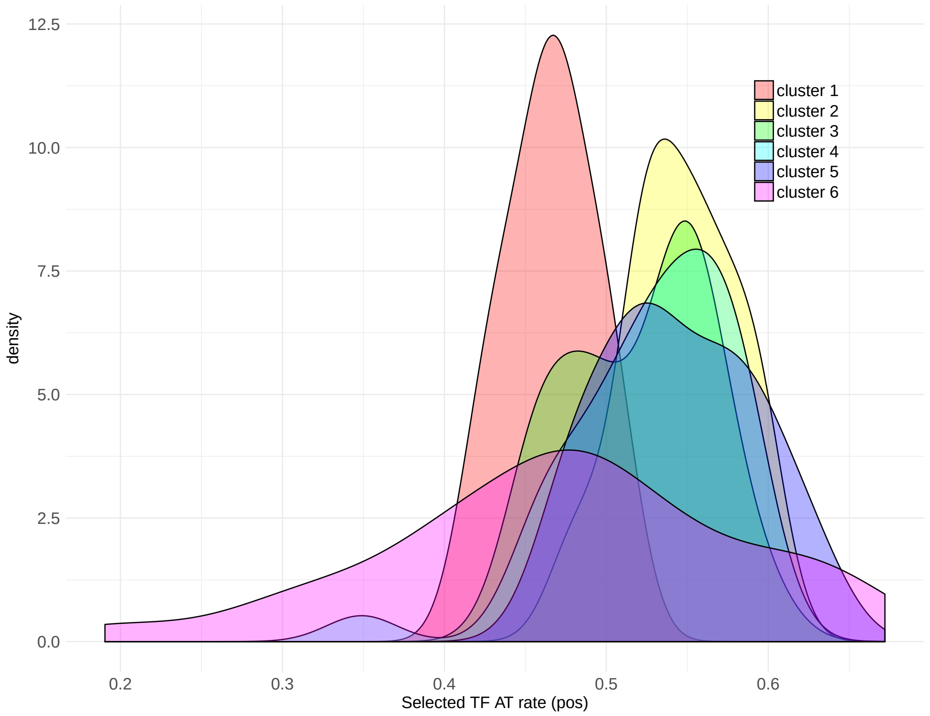

Figure S20. AT rate distributions of selected PWMs in enhancer models (with $\beta > 0$). For each cluster we keep one model per target PWM to avoid bias due to overrepresentation of some PWMs.

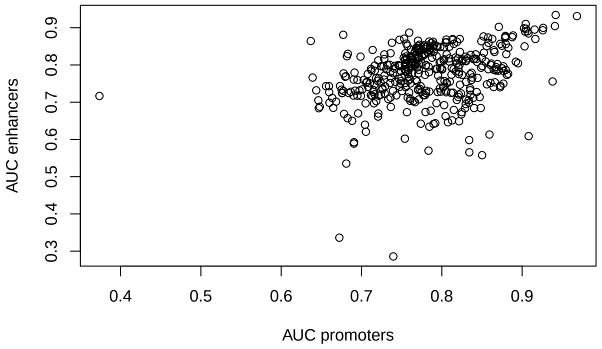

Figure S21. Dotplot of the AUCs computed on mRNA promoter and on enhancers for the same ChIP-seq experiment.
